# What controls the remobilization and deformation of surficial sediment by seismic shaking? Linking lacustrine slope stratigraphy to great earthquakes in South–Central Chile

**DOI:** 10.1111/sed.12856

**Published:** 2021-05-06

**Authors:** Ariana Molenaar, Maarten Van Daele, Thomas Vandorpe, Gerald Degenhart, Marc De Batist, Roberto Urrutia, Mario Pino, Michael Strasser, Jasper Moernaut

**Affiliations:** ^1^ Institute of Geology University of Innsbruck Innrain 52, 6020 Innsbruck Austria; ^2^ Renard Centre of Marine Geology Ghent University Sint‐Pietersnieuwstraat 33, 9000 Ghent Belgium; ^3^ Flanders Marine Institute (VLIZ) Wandelaarkaai 7, 8400 Oostende Belgium; ^4^ Department of Radiology Core facility Micro CT Medical University of Innsbruck Christoph‐Probst‐Platz 1, Innrain 52 A, 6020 Innsbruck Austria; ^5^ Faculty of Environmental Sciences EULA‐Center University of Concepción Barrio Universitario s/n Concepción Chile; ^6^ Inst. Ciencias de la Tierra Transdisciplinary Center for Quaternary Research in the South of Chile Universidad Austral de Chile Valdivia Chile

**Keywords:** Chilean subduction zone, palaeoseismology, soft sediment deformation, surficial remobilization, turbidite

## Abstract

Remobilization and deformation of surficial subaqueous slope sediments create turbidites and soft sediment deformation structures, which are common features in many depositional records. Palaeoseismic studies have used seismically‐induced turbidites and soft sediment deformation structures preserved in sedimentary sequences to reconstruct recurrence patterns and – in some cases – allow quantifying rupture location and magnitude of past earthquakes. However, current understanding of earthquake‐triggered remobilization and deformation lacks studies targeting where these processes take place, the subaqueous slope and involving direct comparison of sedimentary fingerprint with well‐documented historical earthquakes. This study investigates the sedimentary imprint of six megathrust earthquakes with varying rupture characteristics in 17 slope sediment cores from two Chilean lakes, Riñihue and Calafquén, and evaluates how it links to seismic intensity, peak ground acceleration, bracketed duration and slope angle. Centimetre‐scale stratigraphic gaps ranging from *ca* 1 to 20 cm – caused by remobilization of surficial slope sediment – were identified using high‐resolution multi‐proxy core correlation of slope to basin cores, and six types of soft sediment deformation structures ranging from *ca* 1 to 25 cm thickness using high‐resolution three‐dimensional X‐ray computed tomography data. Stratigraphic gaps occur on slope angles of ≥2.3°, whereas deformation already occurs from slope angle 0.2°. The thickness of both stratigraphic gaps and soft sediment deformation structures increases with slope angle, suggesting that increased gravitational shear stress promotes both surficial remobilization and deformation. Seismic shaking is the dominant trigger for surficial remobilization and deformation at the studied lakes. Total remobilization depth correlates best with bracketed duration and is highest in both lakes for the strongest earthquakes (*M*_w_ *ca* 9.5). In lake Riñihue, soft sediment deformation structure thickness and type correlate best with peak ground acceleration providing the first field‐based evidence of progressive soft sediment deformation structure development with increasing peak ground acceleration for soft sediment deformation structures caused by Kelvin‐Helmholtz instability. The authors propose that long duration and low frequency content of seismic shaking favours surficial remobilization, whereas ground motion amplitude controls Kelvin‐Helmholtz instability‐related soft sediment deformation structure development.

## INTRODUCTION

Turbidites and soft sediment deformation structures (SSDS) are ubiquitous within many sedimentary records (turbidites: e.g. Bouma, 1962; Lowe, 1982; Mutti, 1992; SSDS: e.g. Lowe, 1975; Allen, 1982; Maltman, 1994). Besides triggers such as storms, tsunamis, floods and rapid sediment loading, also earthquakes are considered a main cause for the formation of these sedimentary features. Several subaqueous palaeoseismological studies on marine and lacustrine sediment archives used earthquake‐triggered turbidites (e.g. Goldfinger *et al.,* 2012; Howarth *et al.,* 2014; Pouderoux *et al.,* 2014; Ikehara *et al.,* 2016; Moernaut *et al.,* 2018) and SSDS (e.g. Marco *et al.,* 1996; Hibsch *et al.,* 1997; Becker *et al.,* 2002; Üner *et al.,* 2019; Lu *et al.,* 2020) to unravel earthquake recurrence patterns and sometimes provide more quantitative characterization of earthquake magnitude and rupture location. Full exploitation of palaeoseismic turbidite and SSDS records hinges on a thorough understanding of the effect of: (i) different ground motion characteristics; (ii) sediment properties; (iii) slope morphology; and (iv) seismic site effects on causative processes like surficial remobilization and deformation. However, there is a lack of detailed studies of where these processes take place: the subaqueous slopes.

Surficial remobilization describes the earthquake‐induced remoulding and subsequent transportation of a centimetre‐scale veneer of surficial subaqueous slope sediment (e.g. McHugh *et al.,* 2016, 2020; Moernaut *et al.,* 2017; Molenaar *et al.,* 2019; Ikehara *et al.,* 2020; Schwestermann *et al.,* 2020). In contrast, submarine landsliding involves metre‐scale sediment packages failing and sliding along geotechnically ‘weak layers’. Whereas surficial remobilization is caused by seismically‐induced transient stresses at the sediment–water interface (Moernaut *et al.,* 2017; Gomberg, 2018), submarine landsliding strongly depends on preconditioning factors, such as build‐up of critical overburden stress, availability of weak layers and excess pore pressure, in addition to a trigger mechanism such as earthquake shaking (Locat & Lee, 2002). Moernaut *et al*. (2017) stated that surficial remobilization could explain the correlation of turbidite volume (or thickness) with seismic shaking intensity observed in some settings (Goldfinger *et al.,* 2012; Moernaut *et al.,* 2014; McHugh *et al.,* 2016), because stronger shaking would remould deeper into the sediment resulting in higher turbidite volume. Therefore, seismically‐induced surficial remobilization might allow for quantification of palaeoseismic intensity through the volumetric analysis of seismo‐turbidites.

Most existing studies inferred surficial remobilization by studying remobilized sediment in trenches and isolated slope basins at active margins or in lacustrine basin floors (e.g. Ashi *et al.,* 2014; McHugh *et al.,* 2016; Moernaut *et al.,* 2017; Kioka *et al.,* 2019a; Ikehara *et al.,* 2020). However, turbidite composition is influenced by a wide range of processes during remobilization, transport and deposition, which can introduce bias to interpretation of the underlying remobilization process (Schwestermann *et al.,* 2020). Recently, surficial remobilization was stratigraphically detected on a Japan Trench slope (2.5° slope angle) in the form of three centimetre‐scale gaps that were temporally linked to the three strongest (*M*_w_ > 8) regional historical earthquakes (Molenaar *et al.,* 2019). However, one slope site does not suffice to evaluate the modulating effect of morphology on surficial remobilization or the spatial extent of centimetre‐scale gaps over extensive slope areas.

Soft sediment deformation structures (SSDS) form when sediment strength is reduced through sudden increase of pore pressure facilitating hydroplastic deformation, liquefaction or even fluidization with increasing pore pressure to grain weight ratio (Knipe, 1986; Ortner, 2007). The SSDS have been linked to seismic shaking in many onshore and offshore settings – both contemporaneous and palaeo – and used to investigate palaeo‐earthquakes and unravel earthquake recurrence patterns (e.g. Marco *et al.,* 1996; Becker *et al.,* 2002; Monecke *et al.,* 2004; Obermeier, 2009; Avşar *et al.,* 2016; Lu *et al* *.,* 2020). Previous studies derived quantitative earthquake information from subaqueous sedimentary records by correlating shaking intensity with specific characteristics of lacustrine SSDS, such as thickness (e.g. Hibsch *et al.,* 1997; Rodríguez‐Pascua *et al.,* 2003) or type (e.g. Sims, 1973; Rodríguez‐Pascua *et al.,* 2010; Lu *et al.,* 2020). Most of these studies focused on SSDS related to prehistorical earthquakes on outcrops of palaeo‐lakes (e.g. Marco *et al.,* 1996; Rodríguez‐Pascua *et al.,* 2010), which inhibits validation of estimated ground motion parameters or magnitude of the causative palaeo‐earthquakes. Other studies are based on numerical simulations (Wetzler *et al.,* 2010) – which use generic input parameters and require validation in natural settings – or identified SSDS using two‐dimensional images of a small amount of sediment cores (e.g. Monecke *et al.,* 2004; Avşar *et al.,* 2016; Lu *et al.,* 2020) – which potentially leaves small‐scale features undetected and hinders the evaluation of spatial SSDS variability.

This study targets a total of 17 slope sites in two Chilean lakes – Riñihue and Calafquén – and compares the sedimentary slope sequences to accurately‐dated basinal turbidite records which were induced by five historical and one prehistorical megathrust earthquake with variable rupture modes and magnitudes (Moernaut *et al.,* 2014). This approach allows a detailed study on the effect of different ground motion characteristics and slope morphology on surficial remobilization and deformation. Surficial remobilization is investigated by identifying centimetre‐scale gaps using detailed multi‐proxy correlation of slope cores to these basin seismo‐turbidite records, and deformation by using high‐resolution X‐ray computed tomography (CT) data to resolve SSDS in three dimensions.

## SETTING

### Regional earthquake history

**Fig. 1:**
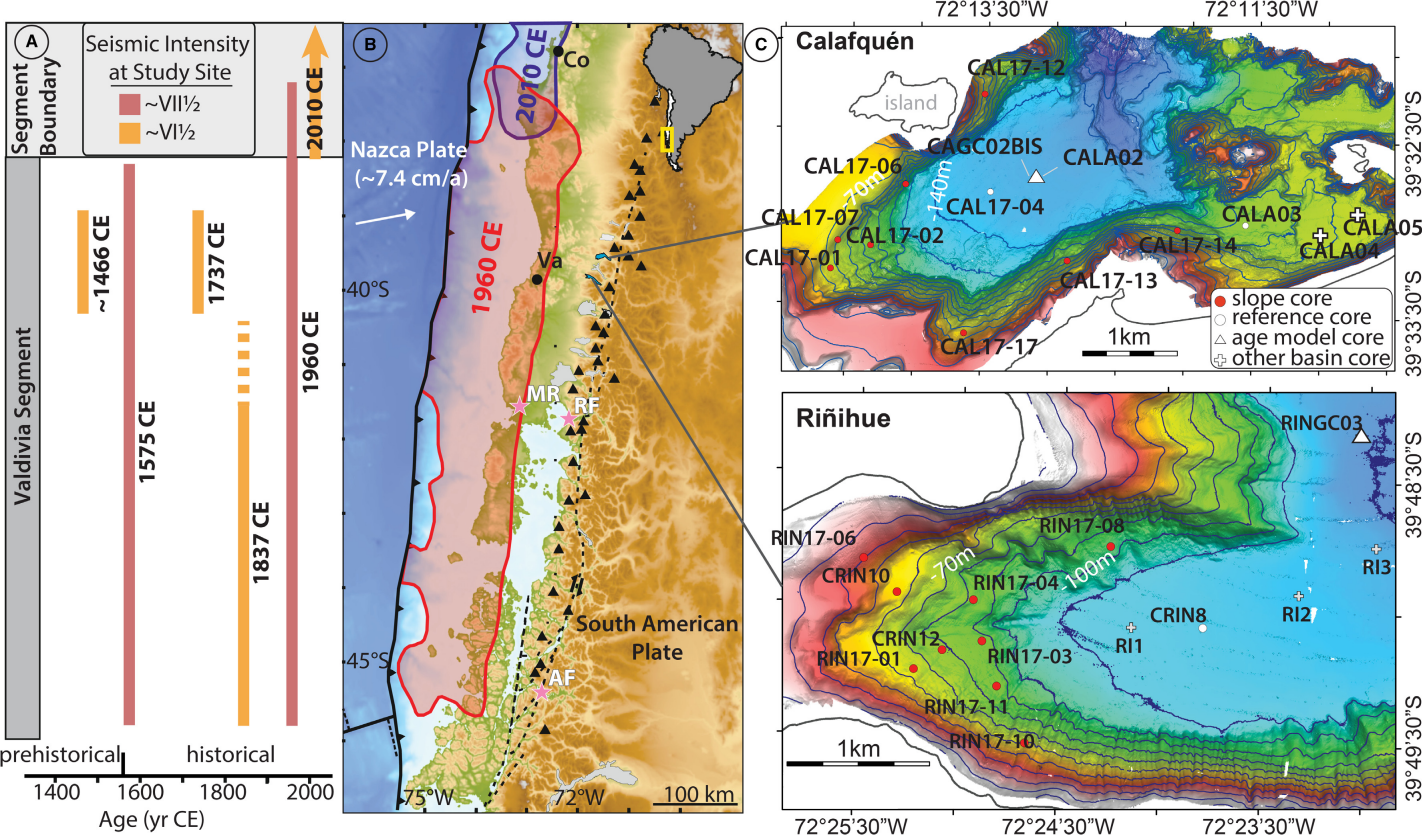
(A) The South–Central Chilean subduction zone and rupture extent of the six studied megathrust earthquakes based on historical and palaeoseismic data (Moernaut *et al.,* 2014; Wils *et al.,* 2020). (B) Tectonic setting of the present study sites and rupture areas (>5 m coseismic slip) of the 1960 ce and the 2010 ce earthquakes (Moreno *et al.,* 2009, 2012, respectively). Triangles depict volcanoes and dashed lines active fault systems. Parts (A) and (B) are adapted from Moernaut *et al*. (2018). Va: Valdivia; Co: Concepción; MR: Maullín River site (Cisternas *et al.,* 2005); RF: Reloncaví Fjord; AF: Aysén Fjord (Wils *et al.,* 2020). (C) Bathymetric maps of the studied basins of lake Calafquén (Moernaut *et al*., 2019) and lake Riñihue (this study) along with the locations of slope cores and reference basin cores as well as previously published basin cores (Moernaut *et al.,* 2014; Van Daele *et al.,* 2014). Contour line distance is 10 m. Full lake bathymetry in Figs S1 and S2.

Lakes Riñihue and Calafquén are located between 39.5°S and 40°S in South–Central Chile, which is tectonically dominated by the subduction of the Nazca Plate below the South American Plate with a convergence rate of *ca* 7.4 cm/a (DeMets *et al.,* 2010; Fig. 1). The subduction zone divides at around 37.5 to 38.5°S into two major seismotectonic segments, the southern Valdivia segment and the northern Maule segment (Métois *et al.,* 2012), both capable of generating giant (*M*_w_ > 8.5) earthquakes. The *M*_w_ 8.8 2010 ce earthquake occurred on the Maule segment and the *M*_w_ 9.5 1960 ce earthquake – the strongest ever recorded – fully ruptured the Valdivia segment. At the Valdivia segment, historical documents describe three other strong historical megathrust earthquakes in 1837 ce, 1737 ce and 1575 ce (Lomnitz, 2004; Cisternas *et al.,* 2005). Another prehistorical earthquake in *ca* 1466 ± 4 ce was identified and accurately dated based on seismo‐turbidite records in lakes Riñihue and Calafquén (Moernaut *et al.,* 2014).

Magnitude and rupture area of the 2010 ce and 1960 ce earthquake are well‐constrained (e.g. Cifuentes, 1989; Moreno *et al.,* 2009, 2012). The 1575 ce earthquake is also assigned to full rupture of the Valdivia segment (*M*_w_ 9.5) because historical reports and geological evidence are strikingly similar to that of the 1960 ce earthquake: similar tsunami and landslide occurrence; turbidite distribution in piedmont lakes, as well as Reloncaví and Aysén fjord; coseismic subsidence and uplift patterns (Cisternas *et al.,* 2005, 2017b; St‐Onge *et al.,* 2012; Moernaut *et al.,* 2014; Wils *et al.,* 2020).

The 1837 ce earthquake is attributed to a wide rupture along the southern half of the Valdivia segment, as suggested by land‐level changes and a large transpacific tsunami (Cisternas *et al.,* 2017a). A minimum magnitude of *M*_w_ 8.8 is considered for the 1837 ce earthquake as tsunami run‐up in Japan and a minimum rupture length of *ca* 500 km – based on historically reported coseismic elevation changes – are within range of the *M*_w_ 8.8 2010 ce earthquake (Cisternas *et al.,* 2017a). Its maximum magnitude of *M*_w_ 9.2 corresponds to the tsunami magnitude assigned by Abe (1979), assuming that tsunami magnitude approximately equals *M*_w_. Possibly, the rupture area of the 1837 ce earthquake extended further north than the northernmost evidence of subsidence, because rather strong shaking, but no damage, was reported in Concepción (Cisternas *et al.,* 2005, 2017a). Using scaling relationships of rupture length versus magnitude proposed by Papazachos *et al*. (2004) and a rupture width of *ca* 200 km (Rotman & Spinelli, 2014; Cisternas *et al.,* 2017a), the authors consider a maximum rupture length of about 620 km to account for a *M*_w_ 9.2 earthquake.

The 1737 ce earthquake was assigned to a narrow and deep rupture along the northern third of the Valdivia segment based on a lack of evidence for a tsunami or coseismic coastal elevation changes, turbidite occurrence at the studied lakes and extent of destruction based on historical witness reports (Moernaut *et al.,* 2014; Cisternas *et al.,* 2017a). A minimum magnitude of *M*_w_ 7.5 was proposed, based on historical reports (Lomnitz, 2004), and a maximum of *M*_w_ 8.2 corresponding to the 1906 ce Valparaíso earthquake, which also ruptured a deeper part of the megathrust (Carvajal *et al.,* 2017; Cisternas *et al.,* 2017a). The prehistorical *ca* 1466 ce earthquake was linked to a similar rupture location and magnitude as the 1737 ce earthquake, based on similar turbidite occurrence and no conclusive evidence of a tsunami or coseismic coastal elevation changes (Moernaut *et al.,* 2014).

For the 2010 ce earthquake, Modified Mercalli Intensities (MMI) at both lakes were estimated as VI^1^/_2_ at lake Calafquén and VI^1^/_4_ at lake Riñihue based on witness reports (Moernaut *et al.,* 2014; U.S. Geological Survey, 2020). For the 1960 ce earthquake, seismic intensity was estimated as VII^1^/_2_ at both lakes using the Medvedev–Sponheuer–Karnik (MSK) scale (Lazo, 2008; Moernaut *et al.,* 2014). For these intensity levels, the MMI and MSK scales give roughly equivalent values (Musson *et al.,* 2010). Accordingly, the general term ‘seismic intensity’ is used here to refer to both scales. Seismic intensity of the other four megathrust earthquakes at the studied lakes was estimated by comparing the cumulative thickness of seismo‐turbidites relative to the 1960 ce and 2010 ce‐related turbidites and further supported by coastal palaeoseismic observations, historically documented geological evidence, as well as earthquake‐related destruction (Moernaut *et al.,* 2014). This results in VII^1^/_2_ for the 1575 ce earthquake and VI^1^/_2_ for the 1837 ce, 1737 ce and *ca* 1466 ce earthquakes.

### Lake setting and sediment

Lakes Riñihue and Calafquén are glacigenic lakes located at the foot of the volcanically active Andes (Fig. 1; Figs S1 and S2). Lakes Riñihue and Calafquén have a size of 28 km × 2–4 km and 24 × 2–6 km and maximum depth of 323 m and 212 m, respectively. Both lakes are oligotrophic and monomictic with mixing during winter (Campos, 1984). The thermocline in both lakes develops from late spring to autumn from *ca* 20 m to *ca* 40 m (Campos, 1984; Campos *et al.,* 2001). No large (>5 m) lake level fluctuations have been reported in historical records, aside from temporary damming of lake Riñihue’s outflowing river by landslides triggered by the 1960 ce and 1575 ce earthquakes (Campos, 1984; Moernaut *et al.,* 2014). The present study focusses on three sub‐basins in the western shallower part of the two lakes (one in lake Riñihue and two in lake Calafquén) sheltered from any major river or lahar inflows to exclude turbidites or erosion related to flood‐induced hyperpycnal flows or the proximal, coarse‐grained fraction of lahars. Earthquake‐triggered turbidites in these basins link to the strong megathrust earthquakes discussed in the *Regional earthquake history* section (Moernaut *et al.,* 2014) and resolved recurrence rates of *ca* 292 years and *ca* 139 years for earthquakes with seismic intensity of ≥VII^1^/_2_ – 1960 ce‐like earthquakes – and ≥VI^1^/_2_, respectively (Moernaut *et al.,* 2018).

The background sediment (i.e. formed during steady‐state sedimentation under normal conditions) of both lakes is continuously varved and consists of millimetre‐scale biogenic varve couplets of diatomaceous ooze related to diatom blooms during spring, and clay to silt sized organic‐rich terrestrial material related to increased run‐off during winter. Basin sequences were accurately dated by varve counting, xs^210^Pb/^137^Cs radionuclide data and tephrochronology (Moernaut *et al.,* 2014; Van Daele *et al.,* 2014). Historically reported volcanic eruptions records match with the age of volcanoclastic layers based on the varve age–depth model in lake Calafquén. This indicates that erosion by fine‐grained basinal turbidites is negligible, thereby ensuring continuity of the varve age–depth model (Van Daele *et al.,* 2014). Background sedimentation rates based on varve counting are *ca* 1 mm/a in both lakes (Moernaut *et al.,* 2014).

Up to 1 cm thick volcanic deposits are embedded in the background sediment and consist of tephra air‐fall deposits and lahar deposits related to volcanic eruptions of nearby volcanoes at the eastern margin of the lakes (Van Daele *et al.,* 2014). Tephra layers are black coarse‐grained deposits corresponding to peaks in magnetic susceptibility. Lahar deposits in the studied basins are light beige or light grey fine‐grained deposits, also corresponding to elevated magnetic susceptibility although less pronounced than the tephra fall deposits. The thickness and grain size of the lahar deposits depends on proximity to the lahar inflow and water depth of the core location as fined‐grained volcanic material is mainly transported by overflows or interflows (Van Daele *et al.,* 2014). Turbidites have a visually homogenous appearance often with a thin coarse‐grained base and thin clay cap, and consist mainly of background sediment within the studied basins (Moernaut *et al.,* 2014; Van Daele *et al.,* 2015).

## METHODS

### Multibeam bathymetry

Multibeam bathymetry of both lakes was acquired in December 2017 using a Norbit WMBS system (wideband multibeam sonar; NORBIT ASA, Trondheim, Norway), combined with an SGR6 (positioning, decimetre horizontal accuracy) and SBD‐IMU‐S2 (roll, pitch and azimuth at <0.02° accuracy). During the survey, pulse frequency as well as swath direction and angle were adapted according to water depth and bottom morphology. Raw data was acquired using QINSy, while processing (sound velocity correction and spike removal) of the data was done using Qimera. The bathymetric maps presented in this study and used for slope angle analysis have a horizontal grid cell size of 2 m. The slope angle of each study site was determined by computing the maximum slope angle at a centre cell based on its eight immediate neighbouring cells using the slope tool from the spatial analyst toolbox in ArcGIS 10.6. For basin sites – where ArcGIS raster‐derived slope angles were inaccurate due to noise within the bathymetric data – slope angles were determined over a 15 m cross‐section perpendicular to the contour lines using Global Mapper 13. To account for possible inaccuracy due to GPS uncertainty, vessel movement, a non‐vertical rope during coring or noise in the bathymetric data, the slope angle over a 12 m, 20 m and 50 m cross‐section perpendicular to the contour lines was measured and used to calculate a maximum and minimum deviation from the ArcGIS‐derived slope angle.

### Sediment cores

Sediment cores were taken at a wide range of water depths (39 to 113 m and 65 to 144 m in lakes Riñihue and Calafquén, respectively) and slope gradients (0.2° to 9.5° and 0.2° to 14.2° in the same lakes) to compare the effect of megathrust earthquakes on slope sites with varying morphology. Sites were chosen on relatively smooth slope morphology away from gullies and at a minimum depth of 39 m to avoid erosion by surface currents and wave action. A water depth that is the same or deeper than the thermocline also allows for deposition of fine‐grained lahar material by interflows (Van Daele *et al.,* 2014) and preservation of millimetre‐scale laminations essential for stratigraphic correlation.

Most sediment cores were taken in December 2017 using a percussion‐driven UWITEC gravity corer (UWITEC, Austria). Reference basin core CALA03 of the south‐eastern basin of lake Calafquén was taken in 2011 and CRIN8 of lake Riñihue in 2015. Before opening, X‐ray computed tomography (CT) scans were taken with a Siemens Somatom Definition Flash (Siemens, Munich, Germany) at Ghent University Hospital with a 0.13 × 0.13 × 0.30 mm resolution. The CT scans of cores CRIN8, CRIN12, CRIN10 and CALA03 were made at the Medical University Innsbruck using a Siemens Somatom Definition AS with 0.20 × 0.20 × 0.30 mm resolution. A section of *ca* 30 cm of RIN17‐11 was cut and µCT‐scanned at a resolution of 0.06 × 0.06 × 0.06 mm using a Scanco Medical XtremeCT II (Scanco Medical AG, Brüttisellen, Switzerland) at the Medical University of Innsbruck. Gamma density of closed cores was measured (0.5 cm step‐size) using a Geotek Multi‐Sensor Core Logger (MSCL; Geotek, Daventry, UK) of the Austrian Core Facility (University of Innsbruck). After core opening, white‐calibrated images were taken using a Smartcube^®^ Camera Image Scanner and magnetic susceptibility was measured (0.2 cm step‐size) using the Geotek MSCL equipped with a Bartington MS2E surface sensor (Bartington Instruments Limited, Witney, UK). Visual contrast and colour variability of the pictures were enhanced using the histogram equalization function in Corel® Photo Paint 2018. This function spreads the most frequent values on the colour intensity histogram generating non‐natural colours to enhance colour variation. Additional non‐white‐calibrated images were made using the camera of a Cox Analytics ITRAX XRF core scanner (Cox Analytical Systems, Gothenburg, Sweden) with polarizing filter to reduce glare of the wet sediment surface. The SSDS were detected and visualized using both FIJI (FIJI is just ImageJ; Schindelin *et al.,* 2012) and VolumeGraphics VGStudio 3.3.

### Ground motion parameters

Peak Ground Acceleration (PGA) and bracketed duration (BD) at the studied lake basins were calculated using empirical ground motion prediction equations developed specifically for the Chilean subduction zone (Idini *et al.,* 2017 and Céspedes *et al.,* 2019, respectively): PGA corresponds to the predicted maximum amplitude of acceleration and BD describes predicted duration of ground motion exceeding ground accelerations of 0.05 g (Kramer, 1996).

Calculated ground motion parameters have large uncertainties because both PGA and BD prediction equations rely on proper estimations of magnitude and rupture extent, which are determined by geological evidence for the pre‐instrumental and prehistorical (i.e. *ca* 1466 ce) earthquakes (*Regional earthquake history* section and Table 1). Magnitude and rupture extent for the 2010 ce and 1960 ce earthquakes are well constrained. For the 1837 ce, 1737 ce and *ca* 1466 ce earthquakes, minimum and maximum magnitudes and corresponding rupture area distances (*Regional earthquake history* section and Table 1) were used to calculate ranges of PGA and BD and corresponding average value. Because both ground motion prediction equations are based on datasets with maximum magnitude of *M*_w_ 8.8 (i.e. ce 2010 earthquake), predicted values for the *M*_w_ *ca* 9.5 1960 ce earthquake and 1575 ce earthquake are less reliable. Because of these uncertainties, the authors do not consider predicted PGA and BD as absolute values, but merely use these values to evaluate correlation of sedimentary imprint (i.e. centimetre‐scale gaps, SSDS and SSDS type) with ground motion parameters.

**Table 1:** Seismic intensity (Moernaut *et al.,* 2014) and predicted ground motion parameters for each megathrust earthquake along with total remobilization depth and soft sediment deformation structure (SSDS) thickness. Predicted peak ground acceleration (PGA) and bracketed duration (BD) values are approximations and should not be considered as absolute values (Ground motion parameters section). Deformation for the 2010 ce earthquake was not considered as top‐most sediment is highly susceptible to coring disturbance. Min. and max. for 1837 ce earthquake correspond to shortest and longest rupture extent, respectively.

Earthquake (ce)	*M*_w_	Coordinates nearest part of rupture	Depth at nearest part of rupture (km)	Closest distance to rupture area (km)		MMI		PGA (g)		BD (s)		Total remobilization depth (cm)		Total EQ‐related SSDS thickness (cm)	
				Riñ	Cal	Riñ	Cal	Riñ	Cal	Riñ	Cal	Riñ	Cal	Riñ	Cal
2010	8.8	38.1°S, 72.9°W	49	201	177	VI 1/4	VI 1/2	0.18	0.20	134	155	0	16	‐	‐
1960	9.5	Riñ: 39.8°S, 72.9°W Cal: 39.5°S, 72.9°W	31	59	59	VII 1/2	VII 1/2	0.57	0.57	1013	1013	43	24	52	0
1837	8.8–9.2	min) 41.5°S, 73.5°W max) 40.4°S, 73.2°W	min) 28 max) 50	212–107	242–139	VI 1/2	VI 1/2	0.17–0.38	0.14–0.31	126–461	107–370	12	0	7	3
1737	7.5–8.2	Riñ: 39.8°S,72.9°W Cal: 39.5°S, 72.9°W	31	59	59	VI 1/2	VI 1/2	0.23–0.35	0.23–0.35	46–135	46–135	0	0	18	30
1575	9.5	Riñ: 39.8°S, 72.9°W Cal: 39.5°S, 72.9°W	31	59	59	VII 1/2	VII 1/2	0.57	0.57	1013	1013	26	28	24	4
*ca* 1466	7.5–8.2	Riñ: 39.8°S, 72.9°W Cal: 39.5°S, 72.9°W	31	59	59	VI 1/2	VI 1/2	0.23–0.35	0.23–0.35	46–135	46–135	0	0	8	32

Ground motion prediction equations for PGA and BD both use shortest distance to the rupture area, magnitude and shear wave velocity in the upper 30 m. The latter was estimated at 200 m/s, which is a characteristic value for soft soils (Ambraseys *et al.,* 1996). The shortest distance from each lake to the fault rupture was calculated by Pythagoras’ theorem using the shortest horizontal distance to the rupture area and the estimated depth of the megathrust seismogenic zone at this location (Tassara *et al.,* 2006).

### Centimetre‐scale gaps

**Fig. 2:**
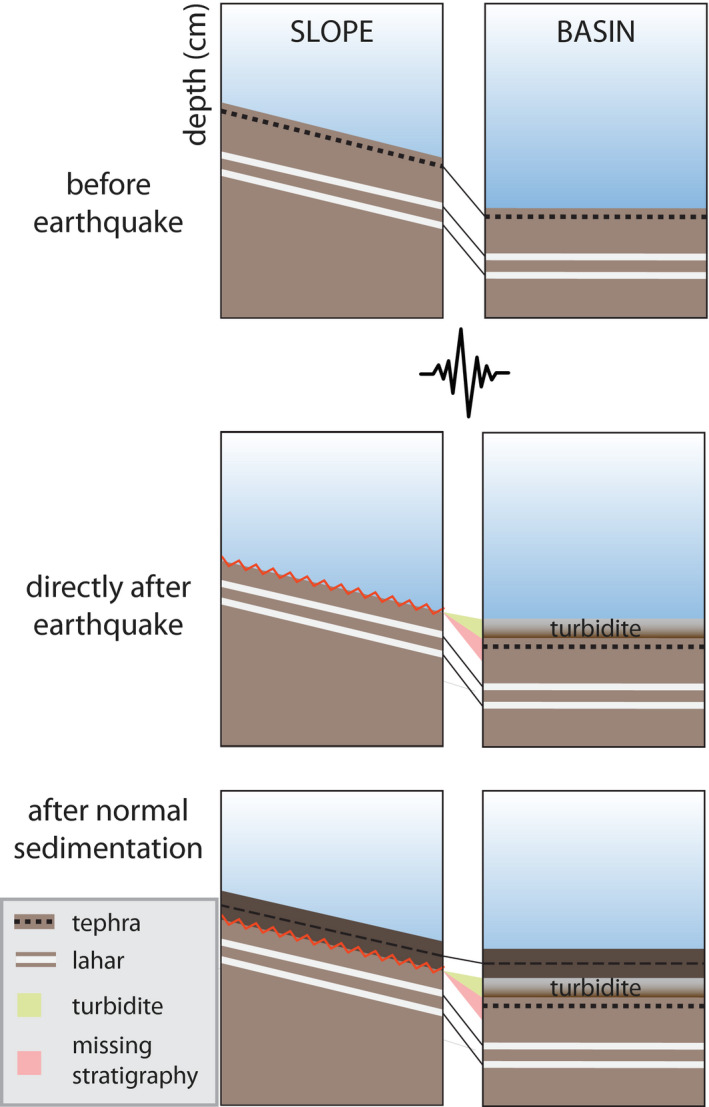
Representation of a slope and basin sequence in three situations: before and directly after an earthquake, as well as after another period of ‘normal’ post‐event sedimentation. Normal sedimentation includes hemipelagic sedimentation intercalated by tephra and lahar deposits.

During an earthquake, the uppermost sediment is eroded from the slope, transported to the basin and deposited as a turbidite (Fig. 2; e.g. Molenaar *et al*. 2019; Schwestermann *et al*. 2020). After the event, normal sedimentation resumes both on the slope and in the basin, creating a gap within the slope sequence exactly at the stratigraphic level of the causative earthquake. Therefore, stratigraphic correlation of slope sequences with continuous basinal sedimentary sequences containing seismo‐turbidites is proposed here as a tool to detect centimetre‐scale gaps potentially caused by earthquake‐triggered surficial remobilization. Gaps are linked to a seismo‐turbidite record within the aforementioned basin core to infer whether these gaps were caused by one of the six megathrust earthquakes.

**Fig. 3:**
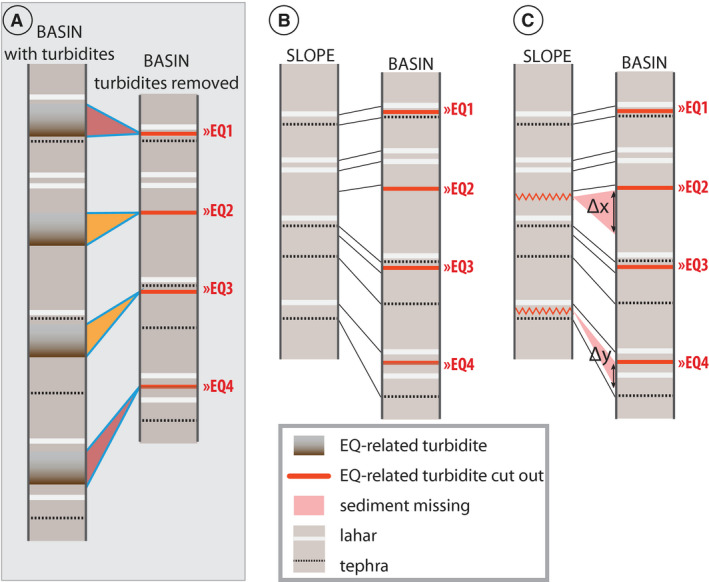
(A) Turbidites are linked to a seismo‐turbidite record and then visually removed from the basin sequence (red line = earthquake marker); (B) and (C) centimetre‐scale gaps are identified and linked to earthquake markers by correlation of a slope sequence to the reference basin sequence without turbidites. Remobilization depth is quantified by measuring the thickness of the missing sediment from the basin sequence (Δ*x* cm and Δ*y* cm).

First, turbidites within a reference basin core are correlated to a seismo‐turbidite record (for example: Figs S3 and S4). Then, to represent a continuous sediment sequence for visual correlations, earthquake‐triggered turbidites are cut from the reference basin sequences and their stratigraphic location marked as ‘earthquake markers’ (Fig. 3A and Fig. S5 as specific examples for this study). Next, the slope sequence is correlated to the corresponding basin sequence by distinct non‐seismic marker layers and trends (for example, tephras, lahar deposits, and variations in magnetic susceptibility or colour) constraining the stratigraphic location of the earthquake marker in the slope sequence by correlating directly above and below this horizon (Fig. 3B). Core correlation is based on images (for example, optical images and CT images) and high‐resolution scanning data (for example, magnetic susceptibility), resolving small‐scale changes in sediment lithology. Finally, gaps are identified in the slope sequence by detecting sediment sections that are present in the basin sequence but not in the slope sequence (Fig. 3C). The amount of remobilization is quantified in centimetres by measuring the thickness of the missing sediment section represented by the basin sequence (Δ*x* and Δ*y* in Fig. 3C). The authors assume that sediment lithology and sedimentation rate on the slope and in the basin are highly comparable if: (i) the study locations are sheltered from major river inlets; (ii) are at or below the thermocline to allow for sedimentation by interflows; and (iii) erosion by turbidites at the basin locations is negligible. Lahar deposit thickness strongly depends on water depth, due to deposition by interflows, and distance to the lahar inflow point (Van Daele *et al.,* 2014). If the thickness of lahar deposits in the studied slope core varies significantly from those in the basin core, remobilization depth is determined from correlation to an intact slope core of comparable water depth.

### Soft sediment deformation structure types

Based on literature, the observed SSDS are subdivided into six types: (i) disturbed lamination; (ii) folds; (iii) intraclast breccia; (iv) faults; (v) load structures; and (vi) injection structures. This study distinguishes between SSDS intervals, which are continuous sections of deformed sediment, and SSDS types as intervals can contain several types of SSDS.

Disturbed lamination involves undulation or thickening and thinning over several laminations without loss of the laminations’ lateral continuity (Rodríguez‐Pascua *et al.,* 2000). Folds describe SSDS where laminations are folded and have a clear vergence. Intraclast breccias are sections of homogenized background sediment comprising fragments of laminations. An intraclast breccia always has a sharp contact to the overlying lamina, but the basal contact can be gradual (Agnon *et al.,* 2006). Normal faults involve brittle deformation of sediment and formation of small‐scale faults. Load structures evolve when overlying sediment sinks into underlying sediment and are mainly driven by inverse density gradients (i.e. denser sediment on top; Owen, 2003). Injection structures form when sediment penetrates into the overlying sediment as pore pressure exceeds lithostatic stress and tensile strength of the overlying sediment (Jolly & Lonergan, 2002).

Coring disturbance can also cause SSDS because: (i) friction at the liner creates domical shapes; (ii) collapse of sediment into gas cracks results in symmetrical normal faults; and (iii) on‐deck core sealing shortly after core acquisition can disturb the top‐most sediment (Jutzeler *et al.,* 2014). Therefore, circular‐symmetrical SSDS; large cylindrical sections of normal faults and disturbed laminations at the core top are interpreted as coring disturbance and excluded from this analysis.

### Sedimentary imprint linked to megathrust earthquakes

Centimetre‐scale gaps are linked to one of the six megathrust earthquakes if correlation lines above and below the gap are also above and below an earthquake marker within the basin sequence. For SSDS intervals, the authors propose one of the six megathrust earthquakes as a trigger if the top of the SSDS interval is within 2 cm below the stratigraphic level of an earthquake, as uppermost sediments are most susceptible to seismically‐induced deformation (Sims, 1973; Marco *et al.,* 1996; Monecke *et al.,* 2004; Avşar *et al.,* 2016).

**Fig. 4:**
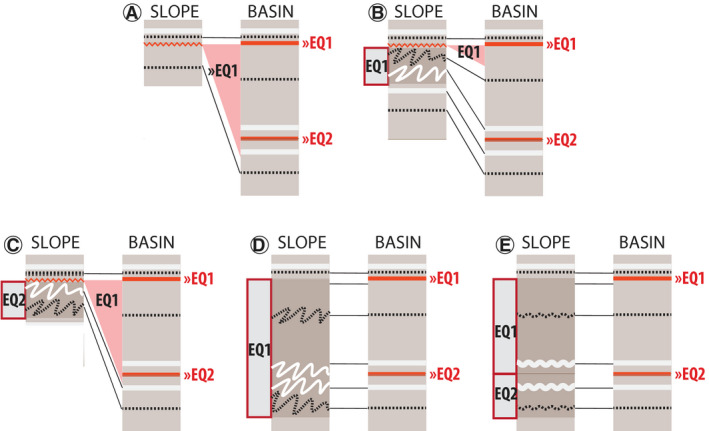
Examples of the correlation strategy herein when centimetre‐scale gaps or soft sediment deformation structures (SSDS) cover the stratigraphical level of multiple earthquake markers. (A) centimetre‐scale gap covering two earthquake markers; (B) centimetre‐scale gap and SSDS correlating to one earthquake marker; (C) centimetre‐scale gap covering two earthquake markers and SSDS; (D) SSDS covering two earthquake markers that deformed the older earthquake marker; (E) SSDS covering two earthquake markers that did not clearly deform the older earthquake marker.

Assumptions must be made when SSDS or gaps cover the stratigraphic level of multiple earthquake markers. In cases where a centimetre‐scale gap eroded the stratigraphic depth of two earthquake markers, it is assigned entirely to the younger earthquake (Fig. 4A). If a SSDS occurs below a gap, the SSDS is correlated to the same earthquake that generated the gap (Fig. 4B), unless the stratigraphic level of another earthquake marker is eroded. In that case, it is verified whether the SSDS was within 2 cm of the sediment–water interface during the older earthquake and the SSDS is correlated to the older event (Fig. 4C). If a SSDS interval covers two earthquake markers within the basin sequence and the stratigraphic level of the older earthquake is clearly deformed – for example with folds or load structures – the SSDS is assigned entirely to the youngest earthquake (Fig. 4D). If a SSDS interval covers two earthquake markers, but the stratigraphic level of the older earthquake is not clearly deformed – for example with disturbed lamination – the SSDS interval is split and assigned to both earthquakes (Fig. 4E). The possible effects of these scenarios are discussed in the *Method evaluation* section.

## RESULTS

### Centimetre‐scale gaps and soft sediment deformation structures in Lake Riñihue and Lake Calafquén

#### Centimetre‐scale gaps

**Fig. 5:**
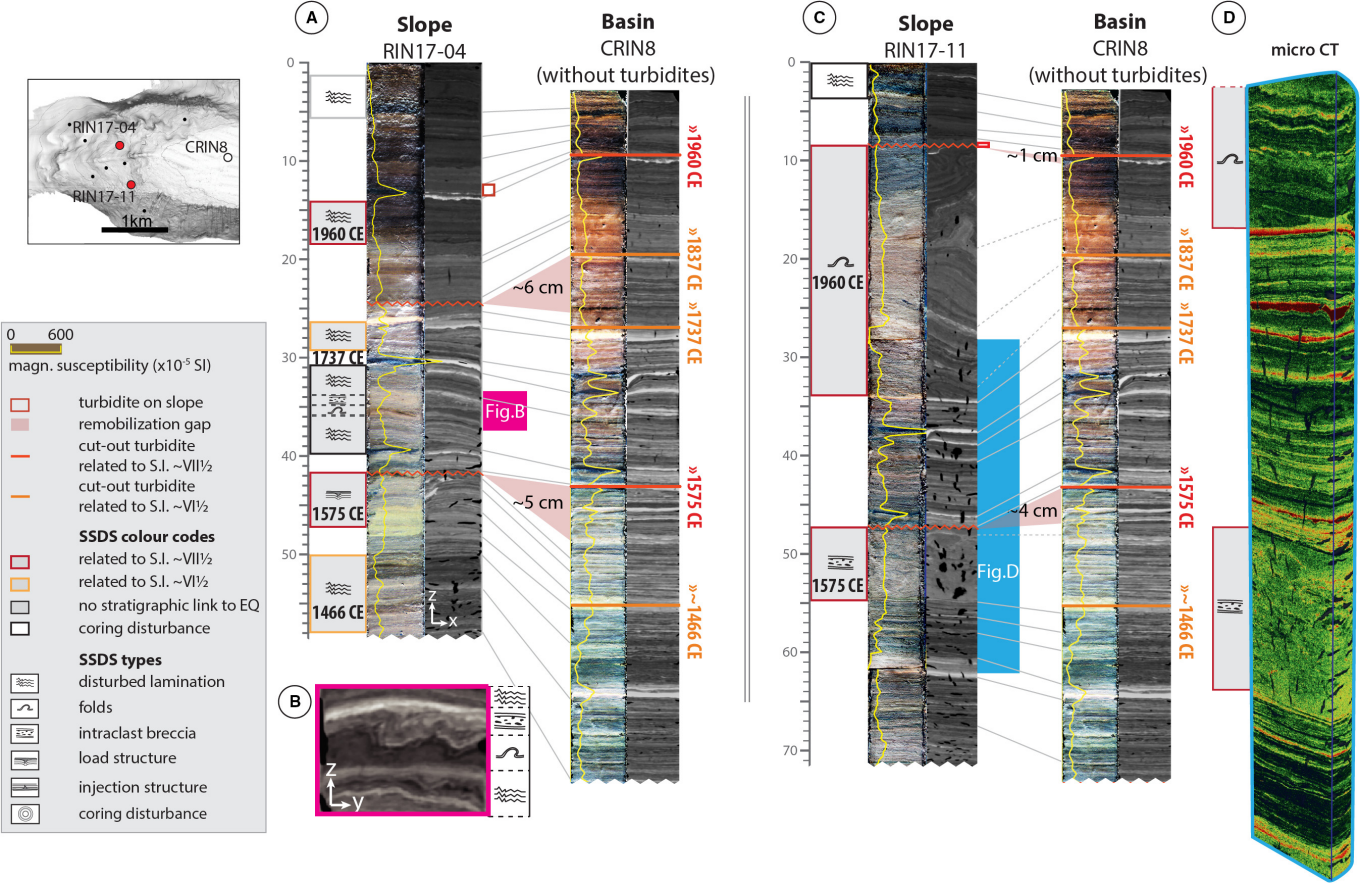
Two examples of slope to basin core correlation (A) and (C) of lake Riñihue and soft sediment deformation structure (SSDS) identification. (B) A SSDS displayed in a CT cross‐section perpendicular (yz‐view) to the orientation of the main CT image (xz‐view); (D) micro‐CT image of RIN17‐11. Micro‐CT colour scale from dark green (low radiodensity) to red (high radiodensity). Figure of the reference basin core with turbidites: Fig. S5. All correlations of lake Riñihue: Figs S7 to S9.

In the lake Riñihue cores, 13 gaps were identified in nine slope sequences with remobilization depths ranging from 1 to 20 cm and average remobilization depth of 6.2 cm (Table 2, Fig. 5 and Figs S7 to S9 for all correlations). These remobilization gaps correspond to *ca* 10 to 233 years missing (average *ca* 60 years) from the slope sequences, as estimated using a sedimentation rate of 1 mm/a (Figs S7 to S9). Undeformed slope sequence thicknesses above or below centimetre‐scale gaps are on average 94% of the reference‐basin sequence thickness, suggesting similar sedimentation rates on slopes and in the basin.

**Table 2:** A summary of earthquake‐related sedimentary imprint in form of: (i) centimetre‐scale gaps represented by remob. (cm) in table; (ii) soft sediment deformation structures (SSDS) thickness represented by deform. (cm) in table; and (iii) SSDS type (DL: Disturbed Lamination; Fo: Folds; IB: Intraclast Breccia; Fa: Faults; LS: Load Structure; IS: Injection Structure). Sedimentary imprint is displayed both per core and per earthquake (i.e. the six megathrust earthquakes). Deformation by the 2010 ce earthquake is not considered due to possible coring disturbance of top sediment.

Lake Calafquén	2010 ce			1960 ce			1837 ce			1737 ce			1575 ce			*ca* 1466 ce			Total remob. per core (cm)	Total deform. per core(cm)
	Remob. (cm)	Deform. (cm)	SSDS type	Remob. (cm)	Deform. (cm)	SSDS type	Remob. (cm)	Deform. (cm)	SSDS type	Remob. (cm)	Deform. (cm)	SSDS type	Remob. (cm)	Deform. (cm)	SSDS type	Remob. (cm)	Deform. (cm)	SSDS type		
CALA03																			0	0
CAL17‐04																			0	0
CAL17‐01																			0	0
CAL17‐02				6															6	0
CAL17‐06	3			6							4	DL	12	3	LS, DL		3	DL	21	10
CAL17‐07	13							3	DL		6	Fo					14	DL, Fo	13	23
CAL17‐12														2	LS				0	2
CAL17‐13				3															3	0
CAL17‐14				7							10	DL, Fo	16				8	DL	23	18
CAL17‐17				2							6	DL							2	6
Total per Lake (cm)	16	‐		24	0		0	3		0	26		28	5		0	32		68	59

Lake Riñihue	Remob.	Deform.	SSDS type	Remob.	Deform.	SSDS type	Remob.	Deform.	SSDS type	Remob.	Deform.	SSDS type	Remob.	Deform.	SSDS type	Remob.	Deform.	SSDS type	Total remob. per core (cm)	Total deform. per core(cm)
CRIN8														1	IB				0	1
CRIN10														2	IB				0	2
CRIN12				6				2	DL					1	IB				6	3
RIN17‐01				20							13	DL, LS, IS	2	1	DL				22	14
RIN17‐03					3	DL								6	Fa				0	9
RIN17‐04					4	DL	6				3	DL	5	5	LS		8	DL	11	20
RIN17‐06				5	7	Fo		4	DL										5	11
RIN17‐08				5	5	DL, Fo	6	1	IB		2	Fo	1						12	8
RIN17‐10				6	8	Fo							14						20	8
RIN17‐11				1	25	Fo							4	8	IB				5	33
Total per Lake (cm)	0	‐		43	52		12	7		0	18		26	24		0	8		81	109
Total per EQ (cm)	16	‐		67	52		12	10		0	44		54	29		0	40		149	168

Thin gravity flow deposits (*ca* 1 cm) occur in slope cores for the 1960 ce (RIN17‐03, RIN17‐04, RIN17‐08, RIN17‐10 and RIN17‐11) and 1575 ce (RIN17‐01, RIN17‐03 and RIN17‐10) earthquakes. These event deposits are identified as turbidites sourced from the higher slope based on their homogenous appearance and thin sandy base in some cases. A thicker gravity flow deposit (3.5 cm) in RIN17‐01 contains lamination fragments indicating a relatively short transport distance compared to the more homogeneous turbidites (Fig. S7).

**Fig. 6:**
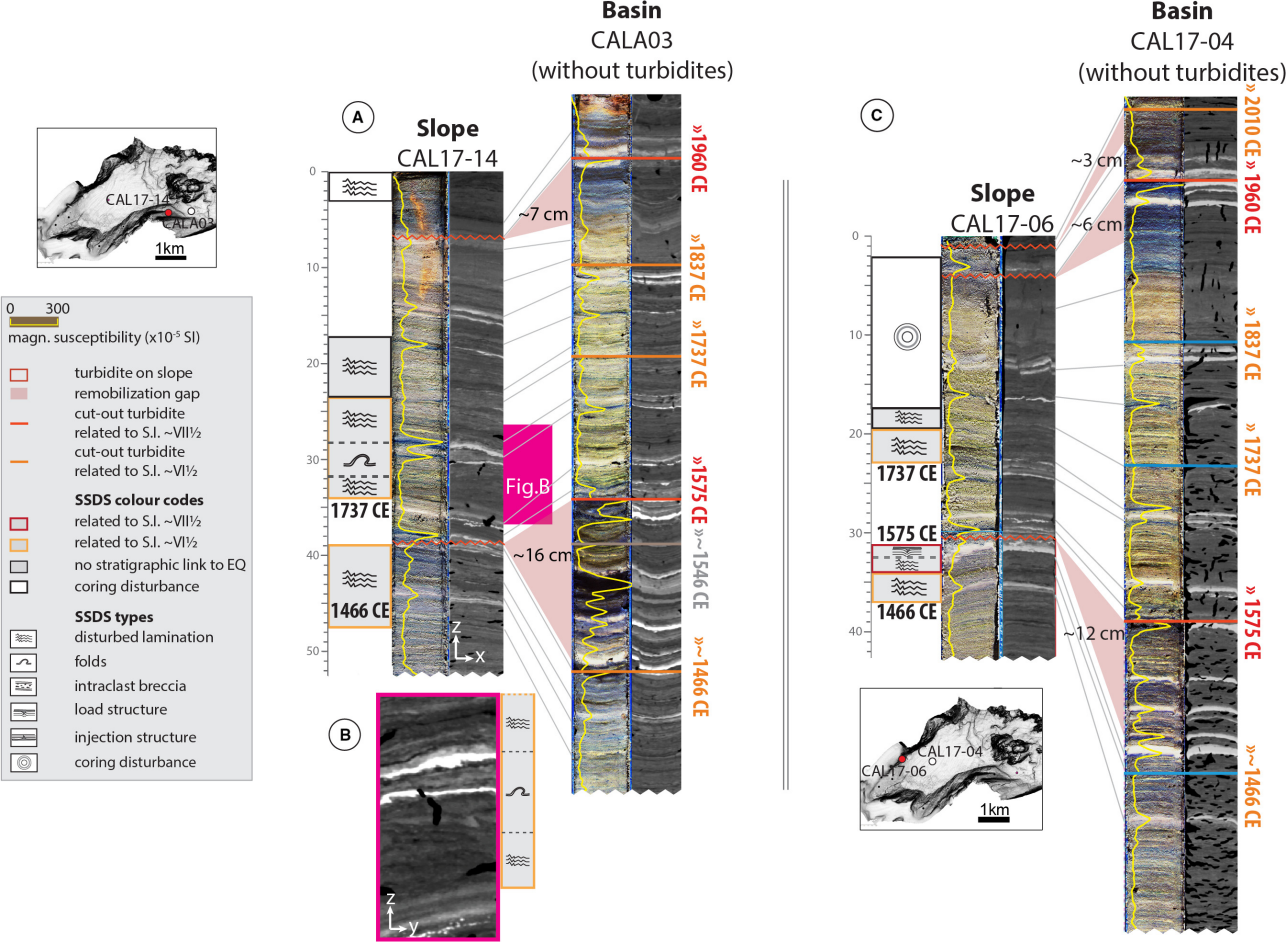
Examples of slope to basin core correlation the western (A) and eastern basin (C) of lake Calafquén. (B) A soft sediment deformation structure (SSDS) displayed in CT cross‐section perpendicular (yz‐view) to the orientation of the main CT image (xz‐view). Figure of the reference basin core with turbidites: Fig. S5. All correlations of lake Calafquén: Figs S10 to S12.

In the lake Calafquén cores, nine gaps were identified in eight slope sequences with remobilization depths ranging from 2 to 16 cm and average remobilization thickness of 7.6 cm (Table 2, Fig. 6 and Figs S10 to S12 for all correlations). These remobilization gaps correspond to *ca* 15 to 120 years (average *ca* 61 years) missing from the slope sequences (Figs S10 to S12) Undeformed slope sequence thicknesses above or below centimetre‐scale gaps are on average 92% of the reference‐basin sequence thickness. Intact slope core CAL17‐01 (water depth 76 m) was used as reference core to determine remobilization depths for the 1960 ce gaps in CAL17‐17 and CAL17‐07 (water depth 65 m and 79 m, respectively) because lahar deposits at this water depth are significantly thinner (*ca* 40%) than in the deeper basin reference core (Figs S10 and S12). Thin (<1 cm) 1960 ce turbidites occur in slope cores CAL17‐02 and CAL17‐17, and 1575 ce turbidites in CAL17‐01 and CAL17‐02.

Cores CAL17‐04 and CALA03 were selected as basin reference cores for the western and eastern basin, respectively (Fig. 6A and C). Aside from turbidites related to the six megathrust earthquakes, CALA03 also includes a single turbidite – dated to approximately 1546 ce – that is not present in any other basin of lakes Riñihue or Calafquén and was likely caused by a local earthquake (Moernaut *et al.,* 2014). In CAL17‐04, another turbidite is located directly above the 1575 ce earthquake with few laminations separating both turbidites. These turbidites are also cut from the basin stratigraphy for comparison to slope sequences.

#### Soft sediment deformation structures

The studied cores of lakes Riñihue and Calafquén contain a total of 31 and 18 SSDS intervals that are not related to coring disturbance, respectively (Figs S7 to S12; *Soft sediment deformation structure types* section). Of these SSDS intervals, 20 in lake Riñihue (i.e. 65% of the total) and 10 in lake Calafquén (i.e. 56% of the total) stratigraphically link to one of the six megathrust earthquakes (Table 2). From the 19 SSDS intervals that do not correlate within 2 cm of an earthquake marker, 10 are located above (i.e. within 1 cm) or within a lahar deposit and four above a tephra layer. Using 3D medical CT scans, SSDS can be identified that would remain undetected in 2D images like split core photographs or radiograph images (examples are Figs 5B and 6B).

**Fig. 7:**
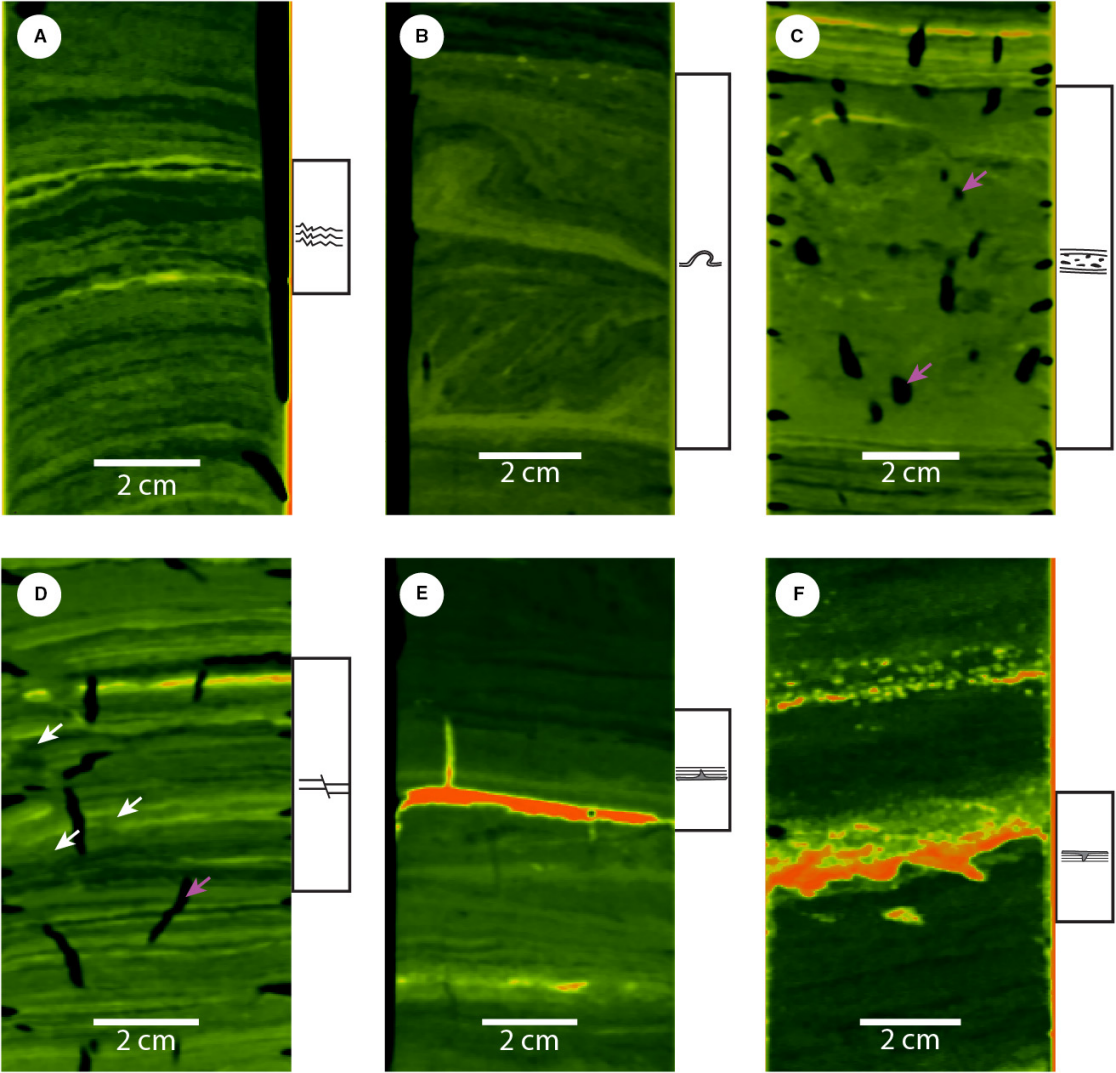
Characteristic examples of the six soft sediment deformation structure (SSDS) types observed in lakes Riñihue and Calafquén obtained from medical CT data (radiodensity: dark green: low; yellow to red: high). (A) Disturbed lamination, (B) folds (white arrows point out offset), (C) intraclast breccia, (D) faults, (E) injection structures, (F) load structures. The colour scale was adapted for each SSDS to optimize visualization. Purple arrows indicate examples of gas cracks caused by degassing of the sediment core.

Cores of lake Riñihue contain all six SSDS types, whereas cores of lake Calafquén do not contain any intraclast breccia (Table 2; Fig. 7). Disturbed laminations are the most common SSDS with 19 and 16 occurrences in lakes Riñihue and Calafquén, respectively. Load and injection structures are scarce with only one injection structure (RIN17‐01) and three load structures (one in lake Riñihue and two in lake Calafquén). Two faults are identified in lake Riñihue (RIN17‐03 and basin core CRIN8). Disturbed laminations are mainly observed in background sediment, whereas folds and intraclast breccia also involve volcanic event layers (i.e. tephra and lahar deposits). Load structures only occur in tephra and lahar deposits, which have higher density than background sediment (Fig. S13). The only injection structure observed in core RIN17‐01 occurred in a tephra layer.

In addition to criteria described in the *Soft sediment deformation structure types* section, intraclast breccias are compared with the corresponding section in the basin sequence to distinguish them from gravity‐flow deposits. Intraclast breccia thickness compares to thickness of the undisturbed basin sequence because they result from *in situ* deformation or only involve very little transportation. Also, intraclast breccias contain clasts of laminations or dispersed high‐density sediment of which the position stratigraphically correlates to the undisturbed basin sequence. Gravity flow deposits also consist of background sediment, but are either homogenous due to complete break‐up of laminae by transport in a turbulent flow (i.e. turbidites) or the original position of lamination fragments is lost during transportation (see RIN17‐01 in Fig. S7). Also, thickness of gravity‐flow deposits can differ largely from the corresponding remobilization depths on the slope (Figs 5 and 6).

Gas cracks hindered the detection of SSDS (especially disturbed lamination and intraclast breccia) in the lower part of cores CAL17‐01, CAL17‐02, CAL17‐17, CAL17‐04 and RIN17‐06. However, if present, the authors expect folds, load structures and injection structures to still be detectable in sediment cores with gas cracks as this involves clear bending and vergence of lamination or high‐density layers. Cores CAL17‐12 and CAL17‐06 contain sections of coring disturbance, and CALA03 is entirely disturbed, due to collapse of gas cracks during core storage. For both lakes, potential deformation by the 2010 ce earthquake is not considered because uppermost sediment is highly susceptible to core disturbance by on‐deck core handling and sealing. Low radiodensity contrast in the upper organic‐rich part of lake Calafquén hindered detection of SSDS for the 1960 ce earthquake, possibly resulting in underestimation of subtle SSDS like disturbed lamination.

### Spatial distribution of sedimentary imprint in lakes Riñihue and Calafquén

**Fig. 8:**
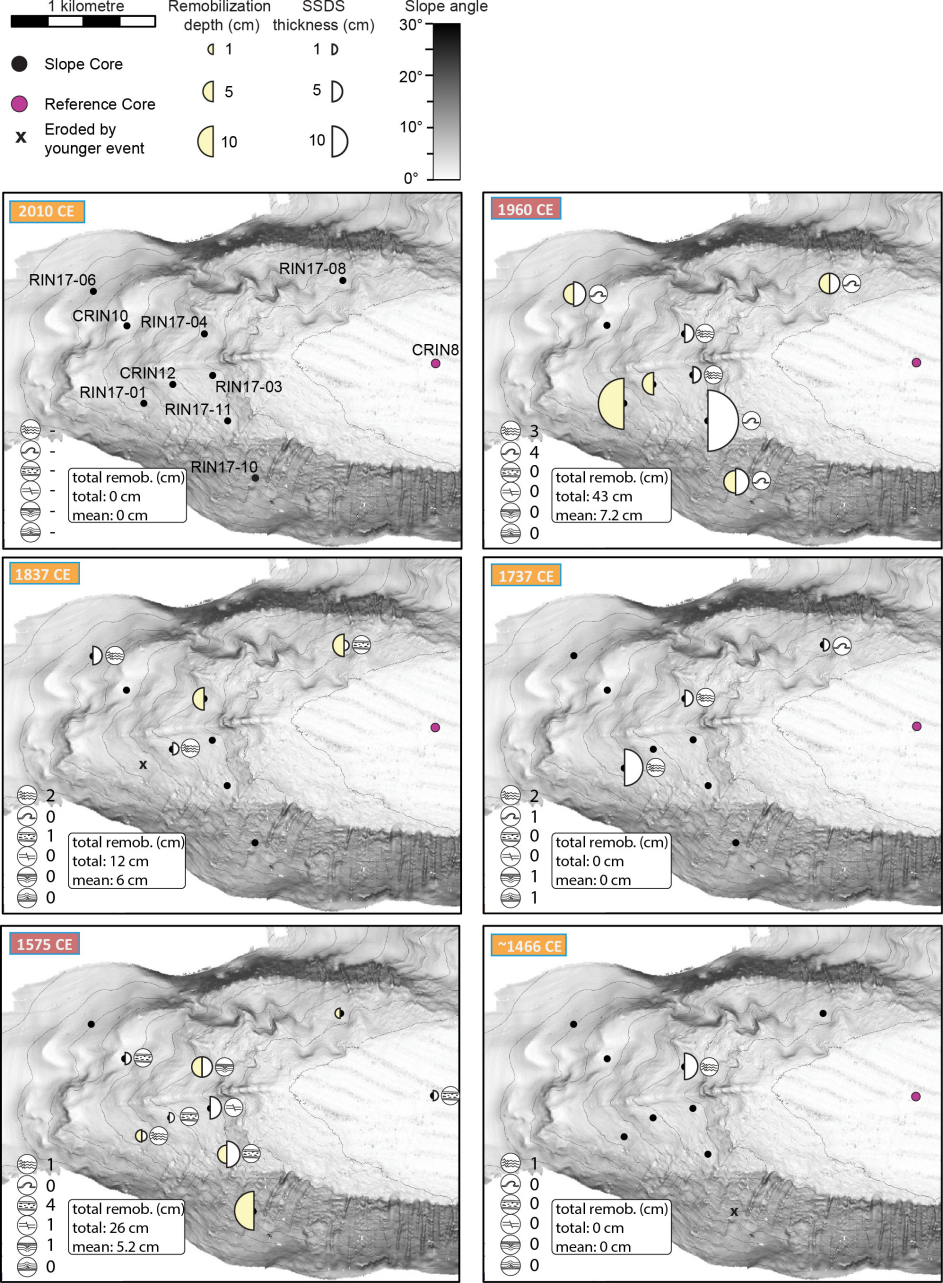
Spatial distribution and thickness of centimetre‐scale gaps and soft sediment deformation structures (SSDS) for the six megathrust earthquakes recorded at lake Riñihue. SSDS type next to core site only depicts the strongest deformation observed for the corresponding earthquake. Below each map is a summary of all SSDS types and total remobilization depth.

For lake Riñihue, eight and nine out of 10 cores contain sedimentary imprint (i.e. centimetre‐scale gaps or SSDS) linked to the 1960 ce and 1575 ce earthquake, respectively (Fig. 8). For the 1837 ce earthquake, centimetre‐scale gaps and SSDS occur predominantly on the northern slope. The 2010 ce, 1737 ce and *ca* 1466 ce earthquakes did not create detectable gaps at the studied core locations. For the 1737 ce earthquake, SSDS occur predominantly on the northern slope. For the *ca* 1466 ce earthquake, one SSDS was identified on the northern slope.

**Fig. 9:**
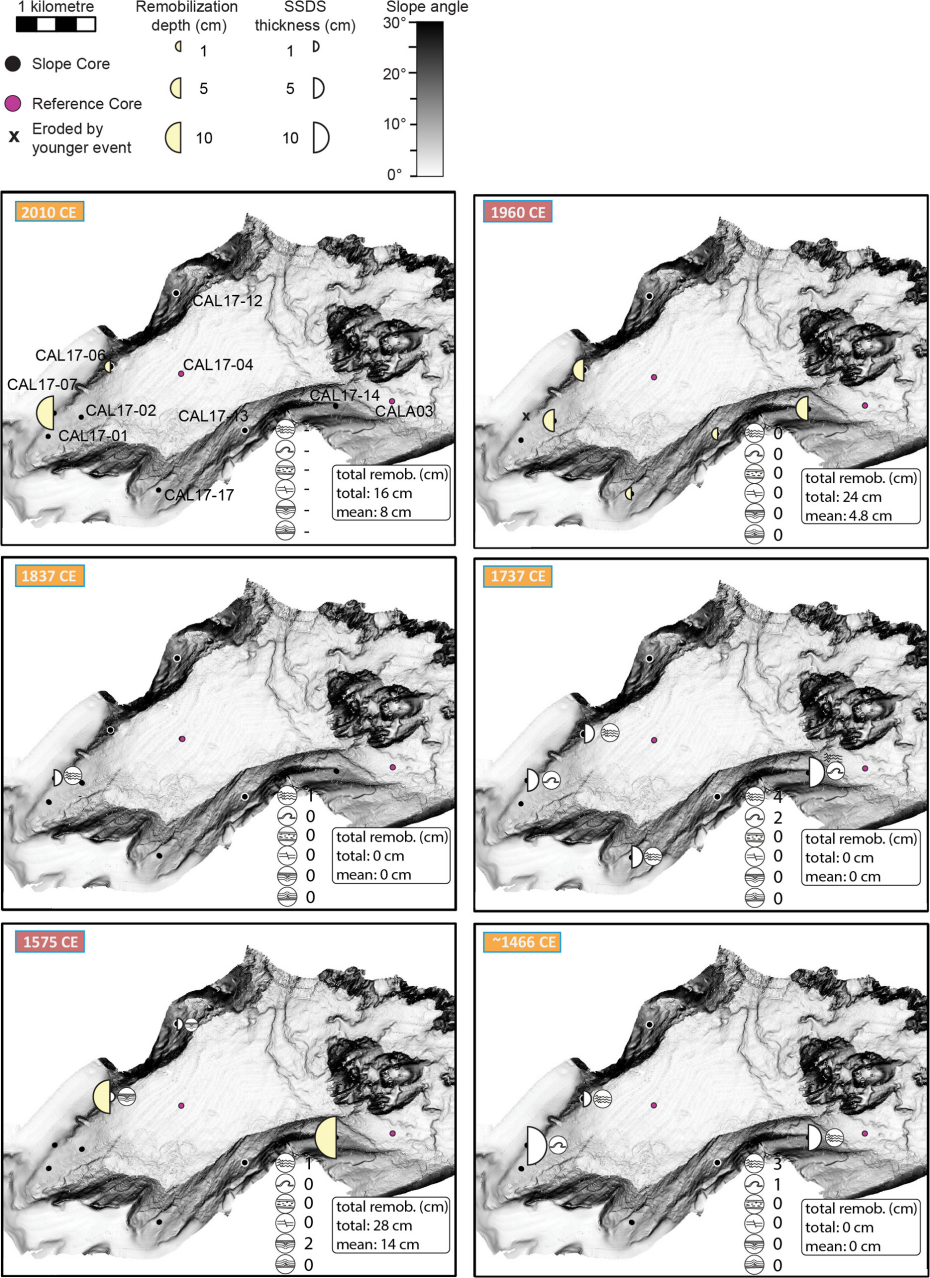
Spatial distribution and thickness of centimetre‐scale gaps and soft sediment deformation structures (SSDS) for the six megathrust earthquakes recorded at lake Calafquén. SSDS type next to core site only depicts the strongest deformation observed for the corresponding earthquake. Below each map is a summary of all SSDS types and total remobilization depth.

In lake Calafquén, centimetre‐scale gaps occur throughout the basin for 1960 ce and two centimetre‐scale gaps – one in the western and one in the eastern basin – can be linked to the 1575 ce earthquake (Fig. 9). For the 2010 ce earthquake, two gaps are located on the north‐western slope. The 1837 ce, 1737 ce and *ca* 1466 ce earthquakes produced SSDS in both the western and eastern basin, but no gaps.

### Total remobilization depth and soft sediment deformation structure thickness versus slope angle

**Fig. 10:**
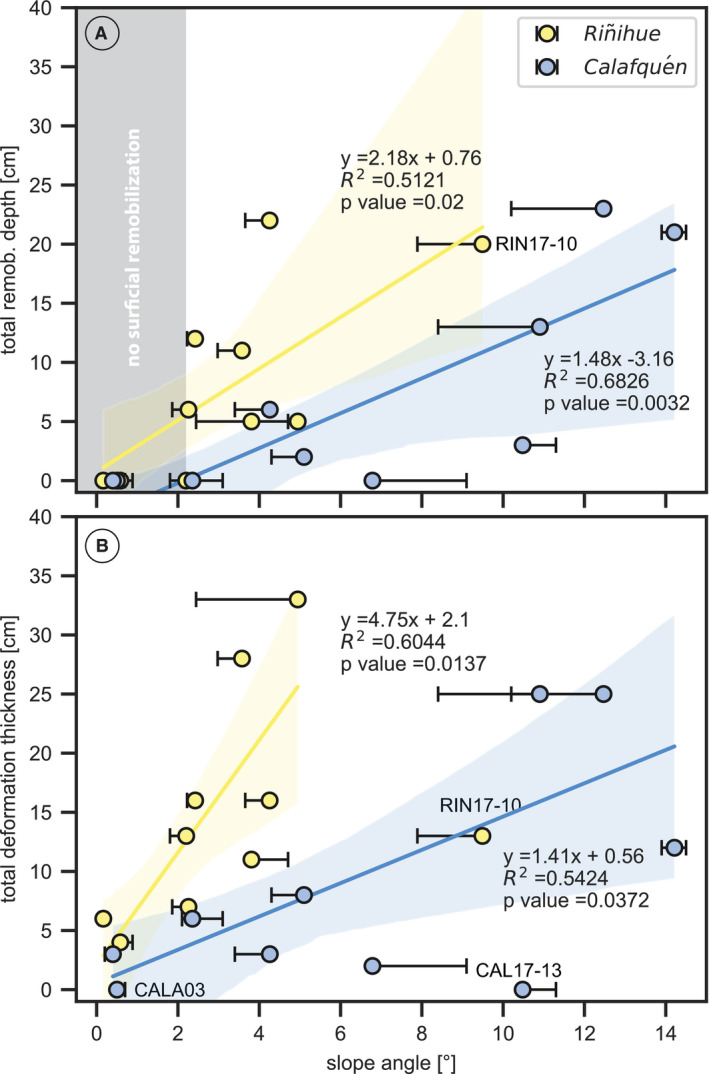
Total remobilization depth and deformation thickness versus slope angle for all core sites along with 95% bootstrap confidence intervals. Total deformation thickness includes both earthquake‐correlated and non‐earthquake‐correlated soft sediment deformation structures (SSDS). Error bars display the maximum and minimum deviation of slope angles measured over 12 m, 20 m and 50 m cross‐sections from the ArcGIS‐derived slope angle.

Total remobilization depth (i.e. cumulative remobilization depth of all centimetre‐scale gaps) and total SSDS thickness per core increase with slope angle in both lakes (Fig. 10). Low (<0.05) *P* values and high (>0.5) *R* ^2^ values for all four correlations statistically support this relationship. The increase with slope angle is stronger in lake Riñihue than in lake Calafquén for both total remobilization depth and total SSDS thickness per core. At the studied sites, centimetre‐scale gaps are only present on slopes with a slope angle ≥2.3°, whereas SSDS also occurs in the basin on slope angles as low as 0.2°.

The minimum and maximum deviation of the slope angle measured over 12 m, 20 m and 50 m cross‐sections from the ArcGIS‐derived slope angle (see *Multibeam bathymetry* section) is represented by the error bars in Fig. 10. A small deviation (i.e. small error bar) indicates that slope angle was rather constant (i.e. for lake Riñihue), whereas a larger deviation indicates that the slope is more convex or concave (i.e. lake Calafquén).

Sites RIN17‐10, CALA03 and CAL17‐13 are treated as outliers in the total SSDS thickness plot and excluded from the linear regression. RIN17‐10 is located between gullies and shows the second highest remobilization depths (i.e. 20 cm in total). Possibly, these high remobilization depths lead to underestimation of total SSDS thickness, as SSDS intervals have been eroded by gravity flows in nearby gullies or earthquake‐triggered surficial remobilization. CALA03 is entirely disturbed by faults related to collapse of gas cracks during core storage. CAL17‐13 has low radiodensity contrast in background sediment inhibiting the detection of disturbed lamination – the most common SSDS in lake Calafquén – leading to a likely underestimation of SSDS in this core.

### Sedimentary imprint compared to ground motion parameters

**Fig. 11:**
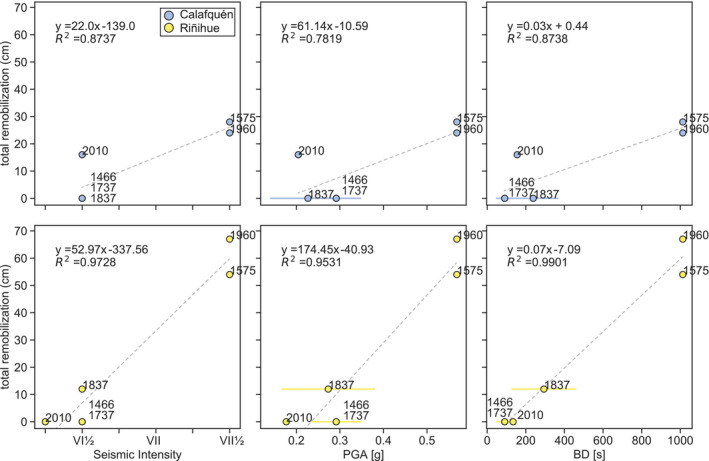
Total remobilization versus seismic intensity, peak ground acceleration (PGA) and bracketed duration (BD) for each of the six megathrust earthquakes as observed in lakes Riñihue and Calafquén. Predicted PGA and BD are used to evaluate correlation of sedimentary imprint with ground motion parameters and should not be considered exact values (*Ground motion parameters* section). Ranges for 1837 ce, 1737 ce and *ca*.1466 ce earthquakes depict minimum and maximum ground motion parameters. Average values of these ranges are used for linear regression.

Total remobilization depth per earthquake correlates best with BD, as predicted using the ground motion prediction equation of Céspedes *et al*. (2019) (*Ground motion parameters* section and Fig. 11). Magnitudes and rupture area distances of pre‐instrumental earthquakes – used to predict ground motion parameters – are based on geological evidence and historical witness reports (*Regional earthquake history* section). The strongest *M*_w_ 9.5 1960 ce and 1575 ce earthquakes with highest predicted BD values caused the largest total remobilization depth (67 cm and 54 cm, respectively) at the studied slope sites of both lakes (Table 2). In lake Riñihue, other centimetre‐scale gaps with a total remobilization depth of 12 cm are detected for the *M*_w_ 8.8 to 9.2 1837 ce earthquake, with second highest predicted BD. In lake Calafquén, the *M*_w_ 8.8 2010 ce earthquake caused a total remobilization depth of 16 cm at the studied sites. The *M*_w_ 7.5 to 8.2 1737 ce and *ca* 1466 ce earthquakes did not create gaps at any of the core sites.

**Fig. 12:**
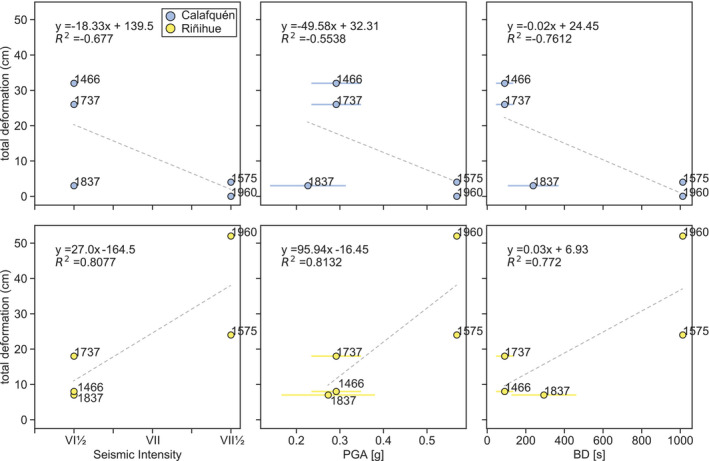
Total deformation versus seismic intensity, peak ground acceleration (PGA) and bracketed duration (BD) for each of the six megathrust earthquakes as observed in lakes Riñihue and Calafquén. Predicted PGA and BD are used to evaluate correlation of sedimentary imprint with ground motion parameters and should not be considered exact values (*Ground motion parameters* section). Ranges for 1837 ce, 1737 ce and *ca* 1466 ce earthquakes depict minimum and maximum ground motion parameters. Average values of these ranges are used for linear regression. Possible causes for negative correlation in lake Calafquén are explained in the *Soft sediment deformation controlled by amplitude of ground acceleration* section.

**Fig. 13:**
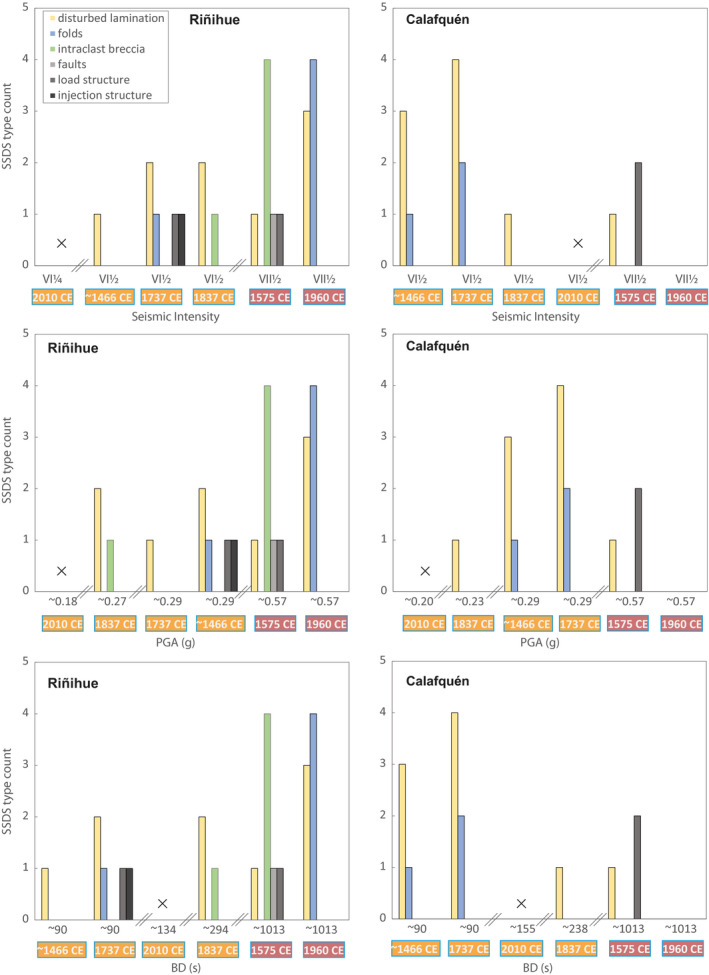
Count of soft sediment deformation structure (SSDS) types for each of the six megathrust earthquakes represented by total remobilization depth and total SSDS thickness depicted for seismic intensity, peak ground acceleration (PGA) and bracketed duration (BD). Single values of BD and PGA of the 1837 ce, 1737 ce and *ca* 1466 ce earthquakes are average values based on magnitude and rupture extent ranges.

At the core sites of lake Riñihue, SSDS count and thickness correlate best with PGA as predicted using the ground motion prediction equation of Idini *et al*. (2017; *Ground motion parameters* section; Figs 12 and 13). Most SSDS were produced during the 1960 ce and 1575 ce earthquakes with highest predicted PGA. Folds are abundant for the 1960 ce earthquake and intraclast breccias for the 1575 ce earthquake, both with four occurrences. The *M*_w_ 8.8 to 9.2 1837 ce earthquake and *M*_w_ 7.5 to 8.2 1737 ce and *ca* 1466 ce earthquakes induced less and thinner SSDS dominated by occurrence of disturbed laminations. At the core sites of lake Calafquén, the 1960 ce and 1575 ce earthquakes with highest predicted PGA relate to no or only three SSDS (i.e. one disturbed lamination and two load structures), respectively. The 1737 ce and *ca* 1466 ce earthquakes caused highest SSDS counts and thickness. Also, the three folds observed in this lake correspond to these two earthquakes – two to the 1737 ce and one to the *ca* 1466 ce earthquake.

## DISCUSSION

### Method evaluation

This proposed method of stratigraphic correlation to well‐dated seismo‐turbidite records proved successful as centimetre‐scale gaps and SSDS were detected in slope sequences and linked to six well‐documented megathrust earthquakes. Precision and detection limit of this novel method hinge on: (i) radiodensity and colour contrasts between background laminations; and (ii) presence of distinct marker layers. High contrast between background lamination and abundant marker layers (for example, flood deposits or tephras and lahars at study sites) ease detection of centimetre‐scale gaps – by improving resolution of stratigraphic correlation – and increase visibility of SSDS. For example, compared to lake Calafquén, detection of gaps and identification of SSDS was easier for lake Riñihue due to higher proxy variation within the sediment. Accuracy of the method relies on similar sedimentation rates at slope and basin sequences as remobilization depths are measured from the basin core sequence (see *Methods – Centimetre‐scale gaps* section). Therefore, basins sheltered from any large local inflows (i.e. river deltas and debris‐flow fans or subaqueous canyons) are ideal. Alternatively, when sedimentation rate or thickness of event layers on slope and basin varies too much, an intact slope core of similar water depth can be used as a reference sequence to determine remobilization depths (for example, cores CAL17‐07 and CAL17‐17 in Figs S10 and S11).

Accuracy also depends on the balance between sedimentation rate and earthquake recurrence as earthquake impact can ‘overprint’ gaps or SSDS created by older earthquakes (Agnon *et al.,* 2006; Molenaar *et al.,* 2019). Overprinting would lead to overestimation of remobilization depth for the younger and underestimation for the older earthquake because gaps are entirely associated with the youngest event (Fig. 4A). In contrast, overprinting of SSDS could lead to underestimation of SSDS thickness for the younger earthquake and overestimation for the older one because this study splits and assigns a disturbed lamination interval correlating to two earthquakes to both the younger and older earthquakes (Fig. 4E). Therefore, high sedimentation rates and long earthquake recurrence intervals enhance the reliability of sedimentary imprint to earthquake assignment as younger earthquakes are less likely to overprint the evidence of older earthquakes.

### Causes of centimetre‐scale gaps and soft sediment deformation structures

#### Are centimetre‐scale gaps related to surficial remobilization?

Aside from seismic shaking, centimetre‐scale erosional gaps in lacustrine sediment sequences may be caused by wave action, lake level fluctuations, seiches or hyperpycnal flows. The minimum water depth of the study sites is 39 m (RIN17‐06) making shallow‐water mechanisms irrelevant, and ruling out wave action and small lake level fluctuation as potential causes of erosion. Large lake level fluctuations (>5 m) have not occurred in these open lake systems located in a temperate rainy climate. Erosion by seismic seiches is dismissed as the erosional impact of seiches would decrease with water depth (Wiegel, 1964) and remobilization thickness does not correlate with water depth. Furthermore, centimetre‐scale gaps occur patchily along the slope (Figs 8 and 9), whereas seiches would rather cause similar bottom current velocity along larger slope areas. Additionally, Van Daele *et al*. (2015) observed that large onshore or offshore landslides are necessary to cause seiches in similar‐sized lake Panguipulli and several smaller lakes in the region. Also, eyewitness reports explicitly mention that no seiching occurred on lake Riñihue during the 1960 ce earthquake (Van Daele *et al.,* 2015), whereas its stratigraphic level shows the largest total remobilization depth in both lakes for all considered earthquakes (Table 2). Only in the south‐eastern end of lake Calafquén (not in the studied basin) some landslide‐induced impulse waves occurred (Sievers, 2000; Van Daele *et al.,* 2015). This demonstrates that seiches are not a prerequisite for surficial remobilization.

The present study sites are located away from any major river or lahar inflow (Figs S1 and S2), thus excluding erosion by any river‐related or lahar‐induced hyperpycnal flows. Erosion related to thin turbidites is negligible on the slope because centimetre‐scale gaps would otherwise occur consistently below turbidites, which is not the case. Also, no erosion was observed for basin floor turbidites (Moernaut *et al.,* 2014; Van Daele *et al.,* 2014) supporting the findings herein of negligible erosion by muddy diatom‐rich turbidites at the study sites (Van Daele *et al.,* 2017).

Gaps at sites RIN17‐01 and RIN17‐10 correspond to highest observed remobilization depths with 20 cm and 14 cm, respectively. Additional erosion due to earthquake‐triggered erosive downslope flows cannot be excluded as these sites are located in the southern gully system (RIN17‐10) and include a 3.5 cm thick 1960 ce‐related event deposit (RIN17‐01). Therefore, these remobilization depths are considered maxima.

All centimetre‐scale gaps correlate to the six megathrust earthquakes, suggesting seismic shaking as the dominant trigger for surficial erosion at the studied slope sites. The authors relate all observed centimetre‐scale gaps to surficial remobilization triggered by seismic shaking, while emphasizing the importance of selecting coring locations sheltered from any gully systems and deep enough to exclude shallow‐water erosive processes.

#### Are all soft sediment deformation structures related to strong seismic shaking?

Earthquakes, but also non‐seismic processes, such as rapid sediment loading and groundwater flow, can trigger deformation of near‐surface sediments (Owen & Moretti, 2011). Wave action is a potential cause of deformation, but can be excluded due to sufficient water depth of the present coring locations (see *Lake setting and sediment* section). Rapid sediment loading by turbidites or high‐density tephra fall is an unlikely mechanism because load structures are not systematically present below such instantaneous deposits: only four load structures occur below tephra deposits and none below turbidites. Also, groundwater movement is ruled out as no pockmarks (i.e. seepage craters) can be observed on the bathymetric data near the study sites. Therefore, the authors propose seismic shaking as the main trigger of SSDS formation at the coring sites in lakes Calafquén and Riñihue.

Most SSDS intervals – 65% and 56% in lakes Riñihue and Calafquén, respectively – were confidently attributed to one of the six strong megathrust earthquakes. It is suggested here that SSDS intervals which are not within 2 cm of the six earthquake markers were either caused by: (i) an unknown local prehistorical earthquake; or (ii) one of the six megathrust earthquakes, which potentially induced dewatering of a deeper tephra or lahar deposit. Dewatering of these volcanic deposits would weaken the overlying sediment and facilitate deformation, a mechanism outlined in Moernaut *et al*. (2019). This latter mechanism may be particularly relevant for the study sites as 13 from 19 SSDS intervals that are not correlated to the six megathrust earthquakes are located above a tephra or lahar deposit.

### How significant is surficial remobilization for earthquake‐triggered sediment transport?

Previous research suggested surficial remobilization to occur uniformly over large slope segments as near‐surface sediments are likely to have similar geotechnical characteristics (Ashi *et al.,* 2014; Moernaut *et al.,* 2017). However, the detailed slope mapping herein reveals that surficial remobilization occurs in a more patchy way as the occurrence and thickness of remobilization along the slopes of the studied basins varies strongly (Figs 8 and 9).

**Fig. 14:**
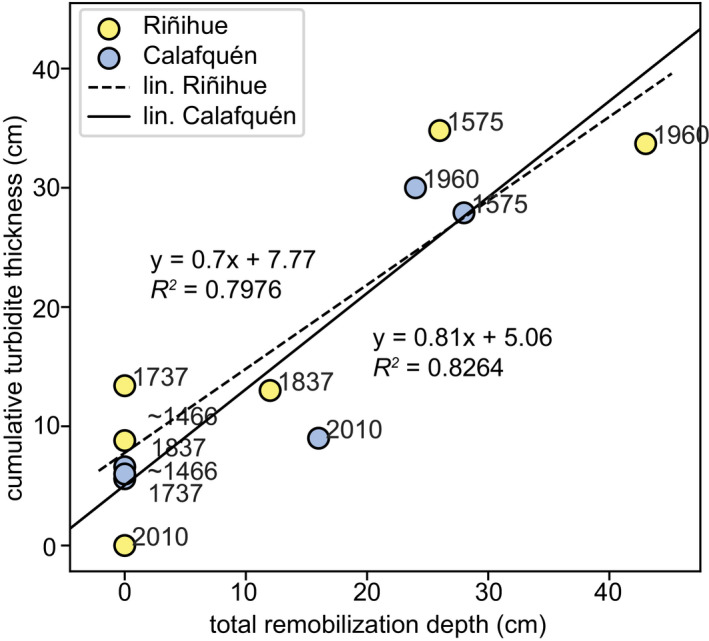
Cumulative turbidite thickness per lake – summing up turbidite thicknesses determined by Moernaut *et al*. (2014) and thicknesses in the reference basin cores herein – versus total remobilization depth (this study) for each of the six megathrust earthquakes.

The remobilization depths inferred from comparison of seismo‐turbidite composition to that of surficial sediments (0.6 to 20.5 cm, average of *ca* 5 cm remobilization depth; Moernaut *et al.,* 2017) are very similar to remobilization depths derived by stratigraphic correlation of slope to basin cores in this study (1 to 20 cm, average of 6.7 cm remobilization depth). Moernaut *et al*. (2017) proposed earthquake‐triggered surficial remobilization as the main process behind turbidite records because volume balance and distribution of mostly small mass‐transport deposits, as well as turbidite composition, ruled out flow disintegration of subaqueous slides as an important turbidite source in these lakes. An exception is the 1960 ce turbidite in lake Calafquén as turbidite composition suggested contamination with older sediments probably linked to an exceptionally large mass‐transport deposit close to the coring site. Moreover, for each event and lake, the cumulative turbidite thickness (Moernaut *et al.,* 2014) correlates well with the total remobilization depth determined in this study (Fig. 14). The 1737 ce and *ca* 1466 ce earthquakes in both lakes and the 1837 ce in lake Calafquén did trigger turbidity currents, which created small turbidites within the basin, but no detectable gaps within the studied slope sequences. Possibly, erosion occurred at steeper slopes, which are more susceptible to erosion (*What is the effect of slope angle on surficial remobilization and deformation?* section), but were not cored during this study. The present study confirms the hypothesis that surficial remobilization is the main remobilization process creating the seismo‐turbidites in the studied basins and not, as commonly assumed, earthquake‐induced slope failures and subaqueous landslides.

At the Japan Trench Margin, surficial remobilization during the *M*_w_ 9.0 2011 ce Tohoku‐oki earthquake eroded and transported sufficient surficial sediment to create widespread seismo‐turbidites with elevated xs^210^Pb activities at hundreds of kilometres along a mid‐slope terrace and in the Japan Trench (McHugh *et al.,* 2016, 2020), moving vast amounts of organic carbon into the trench (Kioka *et al.,* 2019a). Recent studies on radiocarbon composition of seismo‐turbidites show that surficial remobilization was also a relevant remobilization process during historical Japan Trench megathrust earthquakes (Kioka *et al.,* 2019b; Ikehara *et al.,* 2020; Schwestermann *et al.,* 2020). Furthermore, Molenaar *et al*. (2019) detected gaps directly on a northern Japan Trench slope (3138 m water depth) as breaks in the exponential decay profile of xs^210^Pb. These show remobilization depths comparable to the present study (i.e. 4 to 12 cm) and correlate with the largest historical earthquakes in the region including the 2011 ce Tohoku‐oki earthquake.

The evidence for earthquake‐triggered surficial remobilization in Chilean lakes and offshore Japan indicates that this is a common and significant transport process during strong seismic shaking. This finding is important for assessing the reliability of turbidite palaeoseismology as surficial remobilization can facilitate continuous seismo‐turbidite records independent of evolving slope stability conditions and landslide occurrence (Moernaut *et al*. 2017).

### What are the driving mechanisms for deformation in lakes Riñihue and Calafquén?

Disturbed laminations, folds and intraclast breccias are the most common SSDS in lakes Riñihue and Calafquén (Fig. 13) and mainly occur in stable stratified (i.e. density increasing with depth) background sediment (Fig. S13). Similar types of SSDS are observed in the Lisan Formation along the Dead Sea (Marco & Agnon, 1995) and were linked to Kelvin‐Helmholtz Instability (KHI; Heifetz *et al.,* 2005): KHI involves deformation through shear stress build‐up along the interface of stable stratified sediment layers of different density and viscosity due to the horizontal velocity difference of these layers during seismic shaking. The authors propose KHI as the dominant driving mechanism of disturbed lamination, folds and intraclast breccia in the studied lakes. These SSDS form directly at or close to the sediment–water interface (e.g. Sims, 1973; Marco & Agnon, 1995; Lu *et al.,* 2021) making them a valuable time marker for past seismic events.

Other SSDS that sporadically occur at the present study sites are – listed from more to less common – load structures, faults and injection structures. Load structures only formed in high‐density material like tephra and lahar deposits that are present as intercalations in the background sediment. Therefore, these structures are explained following Rayleigh‐Taylor instability, a mechanism driven by inverse density gradients (Owen, 2003). Faults are rare in the studied lakes with two occurrences in RIN17‐03, related to the 1575 ce earthquake, and in CRIN8, not stratigraphically linked to one of the six megathrust earthquakes. Faults form if strain rates during seismic shaking exceed the yield strength of elastically‐behaving sediment (Owen, 1987), and have been reported and linked to earthquakes in different lithologies and settings (e.g. Becker *et al.,* 2002; Monecke *et al.,* 2004; Rodríguez‐Pascua *et al.,* 2010). The only injection structure developed above a tephra layer below a 1960 ce‐related event deposit (RIN17‐01; Fig. S7). Sudden loading by the overlying event deposit may have enhanced pore pressure and simultaneously acted as a lower‐permeability layer, thereby increasing pore pressure while inhibiting immediate pore pressure dissipation. Load and injection structures are – in first order – controlled by availability of density contrasts and liquefaction potential of available sediment (Owen, 2003). Therefore, these SSDS do not necessarily occur at the sediment–water interface (Rodríguez‐Pascua *et al.,* 2000), hampering correlation to specific earthquakes and reducing their palaeoseismological value in the study sites.

### What is the effect of slope angle on surficial remobilization and deformation?

Both remobilization depth and SSDS thickness increase with slope angle at the studied slope sites of lakes Riñihue and Calafquén (Fig. 10), suggesting gravity as a key driving mechanism for surficial remobilization and deformation. Gravitational downslope stress increases with slope angle, thereby enabling downslope movement of remobilized and plastically‐deforming sediment. Increased surficial remobilization at higher slope angles – higher than the maximum 9.5° for lake Riñihue and 14.2° for lake Calafquén – might cause significant erosion of SSDS intervals, potentially leading to underestimation of SSDS thickness at these slope angles.

Surficial remobilization only occurs from a ≥2.3° slope angle, which seems to provide the minimum required gravitational stress at the study sites to enable sediment remobilization and downslope transportation. In contrast, SSDS are present in the nearly flat basin at slope angles of only 0.2° suggesting that seismically‐induced shear stress alone can suffice to deform sediment. Previous research on folded layers within the Lisan Formation reported SSDS at slope angles of <1° and related vergence of folds to the palaeo‐slope direction (Alsop & Marco, 2012, 2013), which demonstrates the high sensitivity of SSDS development to small variations in gravitational downslope stress.

For lake Calafquén, remobilization depth and SSDS thickness increase less with slope angle than for lake Riñihue. This could be due to a higher diatom content in the background sediments of lake Calafquén, illustrated by a combination of lower magnetic susceptibility and abundant bright green laminations identified as diatom blooms (Moernaut *et al.,* 2014; Van Daele *et al.,* 2015). Through high particle interlocking and surface roughness, sediments’ shear strength increases with diatom content (Wiemer & Kopf, 2017) resulting in lower erodibility and lower susceptibility to shear‐induced deformation.

Deformation through KHI nucleates within the uppermost sediment by shear stress at the interface of different sediment layers (Heifetz *et al.,* 2005), whereas surficial remobilization is proposed to initiate at the sediment–water interface due to shear stress between the shaken sediment and the stable water body (Moernaut *et al.,* 2017; Gomberg, 2018). This implies that these are separate processes during the initial stage of seismic shaking. However, during prolonged shaking, increased pore pressure in the deforming sediment would decrease shear strength and may consequently ease erosion through surficial remobilization.

### Which ground motion characteristics control surficial remobilization and deformation?

#### Surficial remobilization controlled by long duration and low frequency content

The occurrence and thickness of centimetre‐scale gaps at studied slope sites in both lakes correlates best with predicted BD, and to a lesser degree with seismic intensity or PGA (Fig. 11). This suggests that surficial remobilization depends more on duration of seismic shaking than on ground motion amplitude. This finding agrees with a recent study by Molenaar *et al*. (2019) at a Japan Trench slope site in which only detectable centimetre‐scale gaps were observed for historical earthquakes of *M*_w_ > 8, despite regional *M*_w_ < 8 earthquakes having PGA values similar or even higher than the *M*_w_ > 8 earthquakes (*ca* 0.6 g). Higher magnitude earthquakes generally correspond to longer shaking duration (Meier *et al.,* 2017), which can explain this observation.

The 2010 ce and 1837 ce ruptures did cause surficial remobilization in lakes Calafquén and Riñihue, despite their large distance to the lakes of 177 km and within 107 to 212 km, respectively. As the high‐frequency ground motion content attenuates strongest with distance, longer travel distances result in ground motion dominated by low frequency components (e.g. Anderson & Hough, 1984). This is in line with observations for the *M*_w_ 9.0 2011 ce Tohoku‐oki earthquake: turbidites triggered by remobilization of surficial slope sediment were identified as far as *ca* 100 km from the rupture area (≥10 m slip; McHugh *et al.,* 2016). Recently, McHugh *et al*. (2020) linked the widespread distribution of 2011 ce turbidites to low‐frequency seismic waves, which would not only attenuate less than short‐period waves, but are also proposed to resonate in the Japan wedge, thereby amplifying and extending the duration of low frequency ground motion.

The findings of this study suggest that long duration and potentially low‐frequency content of ground motion – typical for megathrust earthquakes – favours surficial remobilization. Surficial remobilization cannot be excluded to take place for earthquakes in intraplate or transform plate boundary settings, but dedicated process‐orientated studies in non‐subduction zone settings are lacking to evaluate this.

#### Soft sediment deformation controlled by amplitude of ground acceleration

For lake Riñihue, occurrence and type of SSDS correlate best with PGA (Figs 12 and 13). The 1960 ce and 1575 ce earthquakes correspond to highest predicted PGA as well as highest count of more developed SSDS (i.e. folds and intraclast breccias). The 1737 ce and 1837 ce earthquakes only caused one detected fold and one intraclast breccia in lake Riñihue, respectively. Numerical models predict that SSDS develop with increasing PGA (Heifetz *et al.,* 2005; Wetzler *et al.,* 2010; Lu *et al.,* 2020) from disturbed lamination, folds (i.e. asymmetrical billows and coherent vortices in Lu *et al.,* 2020) and finally intraclast breccia at PGAs of 0.13 g, 0.18 g and 0.54 g, respectively (Lu *et al.,* 2020). Despite very different lithology, the present observations in Chile correspond very well with this PGA to SSDS relationship as disturbed lamination was observed from a minimum of *ca* 0.17 g due to the 1837 ce earthquake, a fold from minimum of *ca* 0.23 g related to the 1737 ce earthquake and abundant intraclast breccias from *ca* 0.57 g induced by the 1575 ce earthquake. Lu *et al*. (2020) demonstrated that geometry of KHI‐driven SSDS also evolves with duration of strong seismic shaking, but PGA values actually regulate the deformation type. However, a direct comparison with the present study is not possible due to a large difference in methodology, for example the timespan of numerical simulations of KHI was significantly shorter (i.e. 1.5 s) than the predicted BD of any of the studied earthquakes (minimum *ca* 46 s; Table 1 and *Ground motion parameters* section).

Based on an unprecedented comparison of SSDS in sediment cores and ground motion parameters of historical earthquakes, this study confirms that the amplitude of ground accelerations is the main seismological control on the occurrence and development of KHI‐related SSDS. Also, PGA is dominated by high‐frequency ground motion (e.g. Kramer, 1996) suggesting that deformation – in contrast to surficial remobilization – is favoured by high‐frequency content. For lake Calafquén, SSDS poorly correlates with seismic intensity, PGA or BD as the strongest 1960 ce and 1575 ce earthquakes caused little deformation. Possible explanations for the lack of SSDS for the 1960 ce and 1575 ce earthquake are that: (i) SSDS for the 1960 ce and 1575 ce earthquakes were underestimated due to lack in radiodensity contrast and abundant gas cracks (*Method evaluation* section); (ii) SSDS for the two strongest earthquakes were eroded by surficial remobilization as, for example, five out of seven slope cores contain 1960 ce‐related centimetre‐scale gaps; or (iii) KHI‐related deformation, which requires stable stratified sediment (i.e. density increasing with depth), was hampered during the 1575 ce earthquake by large density contrasts associated with a high‐density tephra layer a few millimetres below the 1575 ce stratigraphic level (Figs 6 and S13).

For the 1737 ce and *ca* 1466 ce earthquakes, no centimetre‐scale gaps were observed at the studied slope sites and these two earthquakes are the only ones that produced detectable folds in lake Calafquén. Similar sedimentary imprint of both earthquakes on the slopes is in line with comparable turbidite distribution at both lakes (Moernaut *et al.,* 2014) and the lack of conclusive evidence for tsunami or coastal elevation changes. Based on these similarities, the *ca* 1466 ce earthquake was assigned to a rupture location and extent comparable to the 1737 ce earthquake (Moernaut *et al.,* 2014). The new findings herein suggest that lacustrine sediments may ‘record’ differences in ground motion characteristics in the form of particular combinations of SSDS and turbidites. The 1737 ce and *ca* 1466 ce earthquakes correspond to a deep partial rupture of the megathrust at the latitude of Valdivia (Cisternas *et al.,* 2017a), which would cause distinctly different ground motion characteristics compared to the shallower (and wider) ruptures of the other four earthquakes, such as higher frequency content (e.g. Herrera *et al.,* 2020).

Studies have described earthquake‐triggered SSDS in a wide range of subaqueous sediments (for example, ocean, lakes, wetlands and lacustrine cave sediments) at both interplate and intraplate settings (e.g. Marco *et al.,* 1996; Matsuda, 2000; Monecke *et al.,* 2004; Sakaguchi *et al*., 2011; Avşar *et al.,* 2016; Salomon *et al*., 2018; Lauterbach *et al*., 2019; Lu *et al.,* 2020; Oswald *et al*., 2021). This broad variation of geodynamic settings shows that deformation can be caused by different ground motion spectra. In agreement with other research, the present study suggests that sufficiently high PGA is a prerequisite for earthquake‐triggered deformation by KHI and that type of SSDS may be used to quantify past shaking strength (Lu *et al.,* 2020).

## CONCLUSION

This study is the first to directly link sedimentary imprint (i.e. centimetre‐scale gaps and soft sediment deformation structures – SSDS) in lake sedimentary slope sequences with the ground motion parameters of well‐documented megathrust earthquakes. The following conclusions are presented:

1. Surficial remobilization and deformation of slope sediments can be detected and linked to seismo‐turbidite records using detailed stratigraphic correlation of slope sediment cores to well‐dated basin sequences.
2. Six strong megathrust earthquakes are identified as the cause of surficial remobilization and deformation in lakes Riñihue and Calafquén, and other possible trigger mechanisms are excluded.
3. Most of the SSDS occurrences – 65% and 56% in lakes Riñihue and Calafquén, respectively – stratigraphically link to one of the six studied megathrust earthquakes. Other SSDS occurrences might be related to: (i) local earthquakes; or (ii) deformation by one of the six megathrust earthquakes induced deeper in the sequence due to earthquake‐triggered dewatering of intercalated volcanic deposits which weakened overlying sediment.
4. Surficial remobilization is the main process of seismo‐turbidite formation in the studied basins, which agrees with what has been inferred for the Japan Trench. This demonstrates that surficial remobilization is a significant sediment transport process capable of moving vast amounts of sediment into terminal basins of both lakes and ocean.
5. Steeper slope angle increases a slope’s susceptibility to earthquake impact due to higher gravitational downslope stress. Surficial remobilization requires a minimum slope angle (i.e. ≥2.3° at the study sites), whereas small‐scale deformation is observed at nearly flat basin sites of <1°.
6. Total remobilization correlates best with bracketed duration (BD). Based on the present observations, the authors propose that long duration and low frequency content of ground motion favour surficial remobilization.
7. This study provides the first field‐based data directly linking progressive development of Kelvin‐Helmholtz instability‐related SSDS with higher peak ground accelerations (PGAs). Because total deformation and SSDS type correlate best with PGA, the authors propose that surficial deformation is most related to the amplitude of ground acceleration.

## Supporting information


**Figure S1.** Lake Riñihue: Overview map of the complete lake adapted from Moernaut *et al*. (2014).
**Figure S2.** Lake Calafquén: Overview map of the complete lake adapted from Moernaut *et al*. (2014).
**Figure S3.** Basin core correlation of reference basin cores CAL17‐04 and CALA03 to cores and age‐depth model as published by Moernaut *et al*. (2014).
**Figure S4.** Basin core correlation of reference basin core CRIN8 to cores and age‐depth model as published by Moernaut *et al*. (2014).
**Figure S5.** Removal of turbidites from the reference basin core for lake Riñihue (A) and the western (B) and eastern (C) of basin lake Calafquén.
**Figure S6.** SSDS identification in the reference basin cores of lake Calafquén and lake Riñihue.
**Figure S7.** South‐western slope to basin core correlations for lake Riñihue.
**Figure S8.** North‐western slope to basin core correlations for lake Riñihue.
**Figure S9.** Southern and northern slope to basin core correlations of lake Riñihue.
**Figure S10.** Western slope to basin core correlations of lake Calafquén.
**Figure S11.** Southern slope to basin core correlations of lake Calafquén.
**Figure S12.** Northern and eastern basin slope to basin core correlations of lake Calafquén.
**Figure S13.** Two lake Riñihue (RIN17‐03 and RIN17‐08) and two lake Calafquén (CAL17‐01 and CAL17‐04) cores with gamma density data (white) as acquired by Geotek multi‐sensor core logger.

## Data Availability

Data is available from the authors upon reasonable request.
