# Supplementary material for: What controls the remobilization and deformation of surficial sediment by seismic shaking? Linking lacustrine slope stratigraphy to great earthquakes in South–Central Chile
Source: Sedimentology. 2021 May 6;68(6):2365–96. doi: 10.1111/sed.12856 (PMC8518804; doi:10.1111/sed.12856)
Supplement: Supplementary file 1 — Figure S1. Lake Riñihue: Overview map of the complete lake adapted from Moernaut et al. (2014). Figure S2. Lake Calafquén: Overview map of the complete lake adapted from Moernaut et al. (2014). Figure S3. Basin core correlation of reference basin cores CAL17‐04 and CALA03 to cores and age‐depth model as published by Moernaut et al. (2014). Figure S4. Basin core correlation of reference basin core CRIN8 to cores and age‐depth model as published by Moernaut et al. (2014). Figure S5. Removal of turbidites from the reference basin core for lake Riñihue (A) and the western (B) and eastern (C) of basin lake Calafquén. Figure S6. SSDS identification in the reference basin cores of lake Calafquén and lake Riñihue. Figure S7. South‐western slope to basin core correlations for lake Riñihue. Figure S8. North‐western slope to basin core correlations for lake Riñihue. Figure S9. Southern and northern slope to basin core correlations of lake Riñihue. Figure S10. Western slope to basin core correlations of lake Calafquén. Figure S11. Southern slope to basin core correlations of lake Calafquén. Figure S12. Northern and eastern basin slope to basin core correlations of lake Calafquén. Figure S13. Two lake Riñihue (RIN17‐03 and RIN17‐08) and two lake Calafquén (CAL17‐01 and CAL17‐04) cores with gamma density data (white) as acquired by Geotek multi‐sensor core logger. [file SED-68-2365-s001.pdf]

magnitude of earthquakes. *Bull. Geol. Soc. Greece*, **36**, 1482.

Pouderoux, H., **Proust, J.-N.** and **Lamarche, G.** (2014) Submarine paleoseismology of the northern Hikurangi subduction margin of New Zealand as deduced from Turbidite record since 16 ka. *Quat. Sci. Rev.*, **84**, 116–131.

Rodríguez-Pascua, M.A., **Calvo, J.P.**, **de Vicente, G.** and **Gómez-Gras, D.** (2000) Soft-sediment deformation structures interpreted as seismites in lacustrine sediments of the Prebetic Zone, SE Spain, and their potential use as indicators of earthquake magnitudes during the Late Miocene. *Sediment. Geol.*, **135**, 117–135.

Rodríguez-Pascua, M.A., **Garduño-Monroy, V.H.**, **Israde-Alcántara, I.** and **Pérez-López, R.** (2010) Estimation of the paleoepicentral area from the spatial gradient of deformation in lacustrine seismites (Tierras Blancas Basin, Mexico). *Quat. Int.*, **219**, 66–78.

Rodríguez-Pascua, M.A., **de Vicente, G.**, **Calvo, J.P.** and **Pérez-López, R.** (2003) Similarities between recent seismic activity and paleoseismites during the late miocene in the external Betic Chain (Spain): relationship by `b'value and the fractal dimension. *J. Struct. Geol.*, **25**, 749–763.

Rotman, H.M.M. and **Spinelli, G.A.** (2014) Remarkably consistent thermal state of the south central Chile subduction zone from 36°S to 45°S. *J. Geophys. Res. Solid Earth*, **119**, 3503–3516.

Schindelin, J., **Arganda-Carreras, I.**, **Frise, E.**, **Kaynig, V.**, **Longair, M.**, **Pietzsch, T.**, **Preibisch, S.**, **Rueden, C.**, **Saalfeld, S.**, **Schmid, B.**, **Tinevez, J.-Y.**, **White, D.J.**, **Hartenstein, V.**, **Eliceiri, K.**, **Tomancak, P.** and **Cardona, A.** (2012) Fiji: an open-source platform for biological-image analysis. *Nat. Methods*, **9**, 676–682.

Schwestermann, T., **Huang, J.**, **Konzett, J.**, **Kioka, A.**, **Wefer, G.**, **Ikehara, K.**, **Moernaut, J.**, **Eglinton, T.I.** and **Strasser, M.** (2020) Multivariate statistical and multiproxy constraints on earthquake-triggered sediment remobilization processes in the Central Japan Trench. *Geochem. Geophys. Geosyst.*, **21**, 1–24.

Sievers, H. (2000) *El maremoto del 22 mayo de 1960 en las costas de Chile, 2a Edición*. Servicio Hidrográfico y Oceanográfico de la Armada de Chile, Santiago, Chile. pp. 80.

Sims, J.D. (1973) Earthquake-induced structures in sediments of van Norman lake, San Fernando, California. *Science*, **182**, 161–163.

St-Onge, G., **Chapron, E.**, **Mulsow, S.**, **Salas, M.**, **Viel, M.**, **Debret, M.**, **Foucher, A.**, **Mulder, T.**, **Winiarski, T.**, **Desmet, M.**, **Costa, P.J.M.**, **Ghaleb, B.**, **Jaouen, A.** and **Locat, J.** (2012) Comparison of earthquake-triggered turbidites from the Saguenay (Eastern Canada) and Reloncavi (Chilean margin) Fjords: implications for paleoseismicity and sedimentology. *Sediment. Geol.*, **243–244**, 89–107.

Tassara, A., **Götze, H.-J.**, **Schmidt, S.** and **Hackney, R.** (2006) Three-dimensional density model of the Nazca plate and the Andean continental margin. *J. Geophys. Res.*, **111**, B09404.

U.S. Geological Survey (2020) M 8.8 - offshore Bio-Bio, Chile. https://earthquake.usgs.gov/earthquakes/eventpage/official20100227063411530_30/executive. Accessed 5 Dec 2020.

Üner, S., **Özsayın, E.** and **Selçuk, A.S.** (2019) Seismites as an indicator for determination of earthquake recurrence interval: a case study from Erciş Fault (Eastern Anatolia-Turkey). *Tectonophysics*, **766**, 167–178.

Van Daele, M., **Meyer, I.**, **Moernaut, J.**, **de Decker, S.**, **Verschuren, D.** and **de Batist, M.** (2017) A revised classification and terminology for stacked and amalgamated turbidites in environments dominated by (hemi)pelagic sedimentation. *Sediment. Geol.*, **357**, 72–82.

Van Daele, M., **Moernaut, J.**, **Silversmit, G.**, **Schmidt, S.**, **Fontijn, K.**, **Heirman, K.**, **Vandoorne, W.**, **De Clercq, M.**, **van Acker, J.**, **Wolff, C.**, **Pino, M.**, **Urrutia, R.**, **Roberts, S.J.**, **Vincze, L.** and **De Batist, M.** (2014) The 600 yr eruptive history of Villarrica Volcano (Chile) revealed by annually laminated lake sediments. *Geol. Soc. Am. Bull.*, **126**, 481–498.

Van Daele, M., **Moernaut, J.**, **Doom, L.**, **Boes, E.**, **Fontijn, K.**, **Heirman, K.**, **Vandoorne, W.**, **Hebbeln, D.**, **Pino, M.**, **Urrutia, R.**, **Brümmer, R.**, **De Batist, M.** and **Trofimovs, J.** (2015) A comparison of the sedimentary records of the 1960 and 2010 great Chilean earthquakes in 17 lakes: implications for quantitative lacustrine palaeoseismology. *Sedimentology*, **62**, 1466–1496.

Wetzler, N., **Marco, S.** and **Heifetz, E.** (2010) Quantitative analysis of seismogenic shear-induced turbulence in lake sediments. *Geology*, **38**, 303–306.

Wiegel, R.L. (1964) Tsunamis, storm surges, and harbor oscillations. In: *Oceanographical Engineering*, pp. 95–127 Prentice-Hall, Englewood Cliffs, NJ. 16

Wiemer, G. and **Kopf, A.** (2017) Influence of diatom microfossils on sediment shear strength and slope stability. *Geochem. Geophys. Geosyst.*, **18**, 333–345.

Wils, K., **Van Daele, M.**, **Kissel, C.**, **Moernaut, J.**, **Schmidt, S.**, **Siani, G.** and **Lastras, G.** (2020) Seismo-Turbidites in Aysén Fjord (Southern Chile) reveal a complex pattern of rupture modes along the 1960 Megathrust Earthquake segment. *J. Geophys. Res. Solid Earth*, **125**, 1–23.

Manuscript received 17 August 2020; revision accepted 1 February 2021

Supporting Information

Additional information may be found in the online version of this article:

Figure S1. Lake Riñihue: Overview map of the complete lake adapted from Moernaut *et al.* (2014).

Figure S2. Lake Calafquén: Overview map of the complete lake adapted from Moernaut *et al.* (2014).

Figure S3. Basin core correlation of reference basin cores CAL17-04 and CALA03 to cores and age-depth model as published by Moernaut *et al.* (2014).

Figure S4. Basin core correlation of reference basin core CRIN8 to cores and age-depth model as published by Moernaut *et al.* (2014).

Figure S5. Removal of turbidites from the reference basin core for lake Riñihue (A) and the western (B) and eastern (C) of basin lake Calafquén.

Figure S6. SSDS identification in the reference basin cores of lakeCalafquén and Lago Riñihue.

Figure S7. South-western ~~slope-to-basin~~ core correlations for lakeRiñihue.

UNCORRECTED PROOF

© 2021 The Authors. *Sedimentology* published by John Wiley & Sons Ltd on behalf of International Association of Sedimentologists., *Sedimentology*

Figure S8. North-western slope-to-basin core correlations for lake Riñihue.

Figure S9. Southern and northern slope to basin core correlations of lake Riñihue.

Figure S10. Western slope-to-basin core correlations of lake Calafquén.

Figure S11. Southern slope-to-basin core correlations of lake Calafquén.

Figure S12. Northern and eastern basin slope-to-basin core correlations of lake Calafquén.

Figure S13. Two lake Riñihue (RIN17-03 and RIN17-08) and two lake Calafquén (CAL17-01 and CAL17-04) cores with gamma density data (white) as acquired by Geotek multi-sensor core logger.

UNCORRECTED PROOF

© 2021 The Authors. *Sedimentology* published by John Wiley & Sons Ltd on behalf of International Association of Sedimentologists., *Sedimentology*
